# Serum Concentration of the Phytohormone Abscisic Acid Is Associated With Immune-Regulatory Mediators and Is a Potential Biomarker of Disease Severity in Chronic Obstructive Pulmonary Disease

**DOI:** 10.3389/fmed.2021.676058

**Published:** 2021-06-08

**Authors:** Quynh Trang Mi Hoang, Van Kinh Nguyen, Herbert Oberacher, Dietmar Fuchs, Esteban A. Hernandez-Vargas, Katrin Borucki, Nadine Waldburg, Jens Wippermann, Jens Schreiber, Dunja Bruder, Priya Veluswamy

**Affiliations:** ^1^Department of Pneumonology, Otto-von-Guericke-University Magdeburg, University Hospital, Magdeburg, Germany; ^2^Infection Immunology Group, Institute of Medical Microbiology and Hospital Hygiene, Health Campus Immunology, Infectiology and Inflammation, Otto-von-Guericke University Hospital, Magdeburg, Germany; ^3^Department of Infectious Diseases Epidemiology, Imperial College, London, United Kingdom; ^4^Institute of Legal Medicine and Core Facility Metabolomics, Medical University of Innsbruck, Innsbruck, Austria; ^5^Institute of Biological Chemistry, Biocenter, Medical University of Innsbruck, Innsbruck, Austria; ^6^Systems Medicine for Infectious Diseases, Frankfurt Institute for Advanced Studies, Frankfurt, Germany; ^7^Instituto de Matematicas, Universidad Nacional Autónoma de México (UNAM), Queretaro, Mexico; ^8^Institute of Clinical Chemistry and Pathobiochemistry, Otto-von-Guericke University, Magdeburg, Germany; ^9^Ambulatory Pneumology Care, Magdeburg, Germany; ^10^Heart Surgery Research, Department of Cardiothoracic Surgery, Otto-von-Guericke University Hospital, Magdeburg, Germany; ^11^Immune Regulation Group, Helmholtz Center for Infection Research, Braunschweig, Germany

**Keywords:** abscisic acid, PPAR-γ, LanCL2, COPD, asthma

## Abstract

COPD and asthma are two distinct but sometimes overlapping diseases exhibiting varying degrees and types of inflammation on different stages of the disease. Although several biomarkers are defined to estimate the inflammatory endotype and stages in these diseases, there is still a need for new markers and potential therapeutic targets. We investigated the levels of a phytohormone, abscisic acid (ABA) and its receptor, LANCL2, in COPD patients and asthmatics. In addition, PPAR-γ that is activated by ABA in a ligand-binding domain-independent manner was also included in the study. In this study, we correlated ABA with COPD-propagating factors to define the possible role of ABA, in terms of immune regulation, inflammation, and disease stages. We collected blood from 101 COPD patients, 52 asthmatics, and 57 controls. Bronchoscopy was performed on five COPD patients and 29 controls. We employed (i) liquid chromatography–tandem mass spectrometry and HPLC to determine the ABA and indoleamine 2,3-dioxygenase levels, respectively; (ii) real-time PCR to quantify the gene expression of LANCL2 and PPAR-γ; (iii) Flow cytometry to quantify adipocytokines; and (iv) immunoturbidimetry and ELISA to measure CRP and cytokines, respectively. Finally, a multinomial regression model was used to predict the probability of using ABA as a biomarker. Blood ABA levels were significantly reduced in COPD patients and asthmatics compared to age- and gender-matched normal controls. However, PPAR-γ was elevated in COPD patients. Intriguingly, ABA was positively correlated with immune-regulatory factors and was negatively correlated with inflammatory markers, in COPD. Of note, ABA was increased in advanced COPD stages. We thereby conclude that ABA might be involved in regulation of COPD pathogenesis and might be regarded as a potential biomarker for COPD stages.

## Introduction

COPD and asthma constitute a major global health burden (1, 2). The prevalence of COPD and asthma is increasing globally, and COPD has been ranked as a third leading cause of death in the world by 2030 according to the estimates by the World Health Organization (3). COPD is a complex chronic lung disease that is characterized by chronic airflow obstruction, small airway obstruction (4, 5), vascular remodeling (6), and mucus overproduction (7) and to a varying extent by pulmonary emphysema, which is characterized by the loss of alveolar integrity, alveolar space enlargement, and pulmonary hyperinflation (8, 9). Asthma is a complex and chronic inflammatory disease that is characterized by airway hyper-responsiveness, variable bronchial obstruction, and tissue remodeling of airways (10). Although patients with asthma or COPD suffer predominately from dyspnea, the clinical picture may be quite different (11).

Cigarette smoking, exposure to toxic gases, and concurrent respiratory infections are causative factors, which are linked to the pathogenesis of chronic inflammation in COPD (12–14), whereas allergens are considered the primary determinant in most patients with asthma (15, 16). Usually, COPD is manifested with advanced age (17, 18), whereas asthma is frequently seen in children but may occur in adults as well, the latter especially in nonallergic asthmatics (19). COPD is classified based on the Global Initiatives for Chronic Lung Obstructive Diseases (GOLD) scoring system (20), whereas asthma is diagnosed based on spirometry to classify the underlying pathophysiology (21, 22) and are treated based on Global Initiatives for Asthma (GINA) guidelines that correlate with disease severity (23–25).

Neutrophil-driven inflammation, activation of macrophages, and TH_1_ responses characterize for the bronchial inflammation in COPD. These inflammatory cells can secrete a plethora of inflammokines such as reactive oxygen species and tissue-degrading enzymes like matrix-metalloproteinases (MMPs) in addition to the pro-inflammatory cytokines IL-1β, IL-8, IL-6, and TNF-α (26–28). Elevated levels of these inflammatory mediators and decreased levels of anti-inflammatory and immune-regulatory mediators such as IL-10 and indoleamine 2,3-dioxygenase (IDO) (29) further boost disease progression in COPD. Increased CRP blood level is associated with exacerbations, hospitalization, and higher risk of mortality in COPD patients (30). Additionally, obesity and fat-tissue-derived inflammatory mediators may influence pathogenesis in COPD (31–33). In general, tracing of COPD-related mediators in the peripheral blood as biomarkers may represent an interesting option to monitor disease severity and progression (33). It is also evident that patients with severe COPD in their advanced stages exhibit reduced systemic inflammatory cells, in terms of decreased frequencies of lymphocytes (34).

Abscisic acid (ABA) is an evolutionarily conserved isoprenoid phytohormone. It was originally identified in plants but is also found in mammals including humans (35, 36). In plants, ABA regulates physiological functions such as response to abiotic stress factors and cross talk with metabolic signaling pathways in order to combat with drought or pathogen attack (37, 38). In mammals, ABA is produced in and secreted by innate immune cells like granulocytes (39) and monocytes (40) and is therefore detectable in human plasma (41). ABA acts on other cells by binding to the peripheral membrane protein lantibiotic synthetase component C-like 2 (LANCL2) (42, 43) as its direct mammalian receptor. Moreover, ABA also activates the peroxisome proliferation-activating receptor (PPAR-γ) in a ligand-binding domain (LBD)-independent mechanism (44). Both LANCL2 and PPAR-γ are expressed in a wide range of immune cells, including granulocytes (43, 45). Moreover, various pulmonary cell types express PPAR-γ (46). The immunological role of PPAR-γ in attenuating inflammation, including airway neutrophilia, is considered well-renowned. In line with this, the potent anti-inflammatory role induced by ABA was shown to be, in part, influenced by the PPAR-γ activation mechanism (47, 48). Although ABA does not directly bind to PPAR-γ, it has been shown that lack of PPAR-γ in immune cells abrogates the ability of ABA to improve insulin sensitivity and obesity-related inflammation (49).

In previous studies, ABA was described to exert immune-suppressive functions as indicated by preventing immune cell accumulation at sites of inflammation and by downregulating the expression of cell adhesion molecules (47, 50), reducing the levels of pro-inflammatory cytokines/chemokines (50) while increasing the levels of anti-inflammatory cytokines (48, 51), and increasing the number of regulatory T cells and the expression of co-inhibitory molecules on T cells (47, 52). According to its well-proposed anti-inflammatory features, ABA was shown to ameliorate atherosclerosis (50), inflammatory bowel disease (47), obesity-related inflammation (51), and influenza-induced pneumonia (48) in experimental animal models (53).

Based on its above-described immunological roles in various inflammatory disorders, we hypothesize that ABA might as well be involved in the pathogenesis of chronic respiratory diseases in humans. Thus, the aim of this study was to characterize the ABA levels in the peripheral blood and the expression pattern of the ABA receptor LANCL2, as well as PPAR-γ on peripheral blood mononuclear cells of COPD patients and asthmatics, and to correlate these data with disease severity and other clinical parameters associated with COPD and COPD-associated comorbidities. Moreover, a statistical model was applied to evaluate whether the levels of ABA in the systemic circulation would represent a suitable indicator defining the severity of COPD.

## Materials and Methods

### Patients and Controls

One hundred one COPD and 52 asthma patients from the Magdeburg county of Germany were recruited at the Department of Pneumonology, Otto-von Guericke University Hospital, and from a specialized outpatient pneumonology practice in Magdeburg, Germany, under the approval of the institutional ethics committee of the Medical faculty of the Otto-von-Guericke University in Magdeburg (study approval number: 127/15). All patients had an established diagnosis of COPD or asthma according to the current clinical and functional GOLD and GINA criteria, respectively. COPD was diagnosed based on a smoking history ≥ 20 pack years, the absence of a previous or current diagnosis of asthma, a FEV1/FVC ration < 70%, and being under therapy. Asthma was diagnosed in patients with a reversible obstructive impairment of ventilation (increase of FEV1 after two puffs of albuterol > 200 ml) and with smoking history < 10 pack years. Furthermore, the mean lung function (FEV1 %) is given for COPD and asthma patients, in Tables 1, 2, respectively. The inclusion criteria for COPD patients were men and women who had confirmed diagnosis of COPD, were above 40 years of age, and all current and past nicotine abuse of at least 20 pack years. Likewise, the inclusion criteria for asthma patients were men and women who had confirmed diagnosis of asthma and were above 20 years of age with nicotine abuse of at least 10 pack years. Current smokers were excluded from the asthma cohort. COPD and asthma patients without informed consent and with comorbidities (such as malignancy, liver dysfunction) as well as pregnant patients were excluded from the study. COPD patients enrolled in our study were treated with β-agonist, muscarinic antagonist, inhaled corticosteroids (ICS), or a combination of all. Furthermore, 57 age- and gender-matched normal controls were recruited.

**Table 1:** Basic characteristics of COPD patients and controls.

**Patient characteristics**	**COPD blood**	**COPD BAL**	**Control Blood**	**Control BAL**
Number of patients (*n*)	*n* = 101	*n* = 5	*n* = 57	*n* = 29
Mean Age (years) (minimum–maximum)	67 (47–86)	66 (72–61)	57.7 (24–77)	58 (27–79)
Gender (*n*)				
(i) Women	35	5	30	14
(ii) Men	66	0	27	15
Mean body mass index (BMI) (minimum–maximum)	28.7 (17.4–56.1)	28.24 (20.8–33.3)		
(i) Normal weight (18.5–24.9 kg/m^2^)	21.9 (18.7–24.8)	20.8		
(i–ii) Normal weight (<20 kg/m^2^)	18.8 (5 patients)			
(ii) Cachexia (<18.5 kg/m^2^)	17.4 (1 patient)			
(iii) Obese (≥25.0 kg/m^2^)	30.9 (25.0–56.1)	30.1 (27.7–33.3)		
GOLD stages (*n*)				
Stage I	12	0		
Stage II	53	2		
Stage III	8	2		
Stage IV	28	1		
Smoking history (*n*)				
Current smoker	32	2		
Never smoker	8			
Ex-smoker	58	2		
Unknown	3	1		
Mean lung function (FEV1) (%) (minimum–maximum)	63.45 (20–90)	62.7 (20.1–64.1)		
Exacerbation (current status)	94% have no exacerbation	100% have no exacerbation		
Comorbidities	C/P/M/G/R/Mu/Oth	C/P/M/R/Oth		

*C, Cardiovascular; P, Pulmonary; M, Metabolic; R, Renal; Mu, Musculoskeletal; Oth, Others*.

**Table 2:** Basic characteristics of asthmatics and controls.

**Sl. no**	**Patient characteristics**	**Asthma blood**	**Normal control**
(1)	Number of patients (*n*)	52	26
(2)	Mean age (minimum–maximum)	52 (25–74)	50 (25–74)
(3)	Gender		
	(i) Women (*n*)	33	17
	(ii) Men (*n*)	19	9
(4)	Asthma classification^*^		
	(I) Mild intermittent	17	
	(II) Mild persistent	7	
	(III) Moderate persistent	12	
	(IV) Severe persistent	13	
	Unknown	3	
(5)	Mean lung function (FEV1) (%) (minimum–maximum)	78.3 (28.5–137)	

* *Classified by German Airway League and German Respiratory Society. Guidelines for diagnosis and treatment of asthma patients (21)*.

Five patients with COPD underwent bronchoscopy to draw bronchioalveolar lavage fluid (BALF), and additional BALF samples were collected from tumor-free parts of the lung from 29 control subjects who were diagnosed with lung cancer, as listed in Table 1. All subjects gave written informed consent. The basic characteristics of the COPD and asthma patients are outlined in Tables 1, 2, respectively. The basic characteristics including age and gender of control subjects (both blood and BALF controls) were also outlined in Tables 1, 2.

### Bronchoscopy and BALF Handling

Bronchoscopy was performed according to the hospital procedure established at the pneumonology unit, Otto-von-Guericke University Hospital, Magdeburg, and as described in Schreiber et al. (54). The collected BALF was centrifuged to pellet the cells, and the liquid part was stored at −80°C until further usage.

### Whole Blood Collection and Serum Isolation

Five to 10 ml whole blood from COPD and asthma patients and respective age- and gender-matched controls was collected into EDTA tubes and was utilized for RNA isolation and subsequent gene expression analyses. Besides, 10 ml of whole blood was collected in separate serum tubes with clot activating gel (Vacutainer/Sarstedt™) and subjected to centrifugation at 3,300 rpm for 5 min to collect serum that was aliquoted and stored at −80°C until further analysis.

### Measurements of Serum Abscisic Acid by Liquid Chromatography–Tandem Mass Spectrometry

ABA serum levels were determined by liquid chromatography–tandem mass spectrometry (LC-MS/MS). One hundred microliters of plasma was prepared for LC-MS/MS analysis by protein precipitation with acetonitrile/methanol (400 μl, v/v = 9/1). After vortexing and centrifugation, 400 μl of the supernatant was evaporated and redissolved in 19 μl water. Analyte-free plasma obtained from treatment with activated charcoal was used as a surrogate matrix for preparing five calibration standards (0.025–6.25 ng/ml). Sample and standards were spiked prior to sample preparation with the internal standard ABA-D6 (0.25 ng/ml). Here, surrogate matrix samples represented negative controls or blanks. Surrogate matrix samples spiked with different concentrations of abscisic acid were used as calibration standards or positive controls. Chromatographic separation was accomplished with an Eksigent 425 LC system employing a Halo C18 column (100 × 0.5 mm, 2.7 μm, Sciex, Framingham, MA, USA) using a gradient of 2–95% acetonitrile in 0.1% formic acid solution within 10 min. Targets were detected on a quadrupole–quadrupole–time-of-flight instrument (TripleTOF5600+, Sciex) using electrospray ionization (ESI) in negative ion mode. The acquisition strategy involved the collection of full-scan MS/MS spectra (precursor ions: *m/z* 263.1 and 269.2) and post-acquisition extraction of analyte-specific fragment ion mass traces (*m/z* 153.0924 ± 0.05 and *m/z* 159.1543 ± 0.05). The injection volume was set to 5 μl. The column temperature was 25°C. The flow rate was set to 20 μl/min.

### Quantification of LANCL2 and PPAR-γ Gene Expression by Real-Time PCR

Whole blood was collected and subjected to total cellular RNA isolation using QIAamp RNA blood mini kit (Qiagen) (catalog # 52304) according to the instruction manual. The eluted RNA was analyzed to measure the concentrations and purity using the NanoDrop spectrophotometer (NanoDrop 1000, ND-1000 V3.8.1). An almost equal concentration of the RNA template (1 μg) was reverse-transcribed with random primers using the first-strand cDNA synthesize kit (Thermo Scientific, Catalog # K1672). This is to achieve the initial adjustment of sample material, leading to comparable cDNA concentrations in each reverse transcription reaction. The cDNA was stored in −80°C until use. qPCR was performed using the components (i) cDNA template, (ii) primers (forward and reverse), (iii) SYBR Green Master Mix, and (iv) qPCR-grade water in the final volume of 25 μL (FastStart Essential DNA Green Master, Qiagen, Cat# 06402712001). The instrument used for qPCR was the Roche Light cycler which was installed with the Roche Light Cycler 480 Software. Samples were run for 40 cycles (95°C for 15 s, 55°C for 1 min, and 72°C for 1 s). All samples were run in duplicates (i.e., technical replicates). No template control (NTC) served as negative controls. The relative gene expression was calculated using Roche Light Cycler 480 Software. The Ct values were calculated using the second-derivate maximum method. The relative expression (X_norm_) of a target transcript within a given sample was calculated using the delta (Δ) Ct method as follows: X_norm_ = (1 ± E)^−(*Ct, target*−*Ct, reference*)^, with E referring to the PCR efficiency of target and reference PCR reaction (E ≈ 1) (55). Calculation was performed using Ct values, averaged over duplicate measurements. Here, β2-microglobulin (β2M) was used as an internal housekeeping gene (reference gene). The sequences of the primers used in this study are *LANCL2* (forward: TCCTGTCCCTTTACCGTCTCACTC and reverse: GCGAATAGGGTCTGTCAGGAATGC), *PPAR-*γ (forward: AGCCTCATGAAGAGCCTTCCAAC and reverse: TGTTCTCCGGAAGAAACCCTTGC), and β*2M* (forward: TGCCGTGTGAACCATGTGACTT and reverse: GCGGCATCTTCAAACCTCCAT) genes.

### Measurements of Indoleamine 2,3-Deoxygenase (IDO) by HPLC

Tryptophan and kynurenine concentrations in serum were determined by reversed-phase HPLC as described earlier (56, 57). Specimens were deproteinized with trichloroacetic acid and were separated on reversed-phase C18 material using 0.015 mol/l potassium phosphate buffers (pH 6.4). Tryptophan was monitored by means of its native fluorescence (Varian ProStar 360, Palo Alto, CA) at 285-nm excitation and 360-nm emission wavelengths. Kynurenine was detected by ultraviolet absorption (Shimadzu SPD-6A, Korneuburg, Austria) at 365-nm wavelength in the same chromatographic run. Finally, kynurenine/tryptophan (kyn/trp) was calculated as an estimate of IDO activity. Here, tryptophan (100 μM) and kynurenine (10 μM) were used as positive controls, whereas blank served as a negative control.

### Measurements of Adipocytokines by FACS-Based Bead Array

Quantification of the human adipokines adiponectin, adipsin, leptin, and resistin in serum of COPD patients and control subjects was performed using the LEGENDplex™ Human Metabolic Panel 1 (Catalog # 740212, BioLegend) according to the instruction manual.

### Measurement of CRP by Immunoturbidimetry

Serum levels of high-sensitivity C-reactive protein (hsCRP) were quantified by turbidimetry (800/570 nm) with a cobas c 311 analyzer (Roche).

### Measurements of Serum-Soluble Proteins by ELISA

Serum levels of pro-inflammatory cytokines, matrix metalloproteinases, and soluble costimulatory molecules were determined by ELISA according to the manufacturer's instructions. The ELISA MAX™ Standard Set for human TNF-α (Catalog # 430201), IL-8 (Catalog # 431501), IL-1β (Catalog #437004), and IL-6 (Catalog # 430501) was purchased from BioLegend. The human MMP-9 DuoSet ELISA kit was purchased from R&D Systems (DY911-05). The human sCD86 ELISA Antibody Pair Set (Catalog # SEK10699) was purchased from Sino Biological. All analyses were done in duplicates.

### Statistical Analysis

The differences between the analytes in COPD patients and controls (two groups) were performed using either the nonparametric Mann–Whitney *U*-test or unpaired Student *t*-test. Further differences between the three variables with subclassifications within COPD patients (three groups) were performed using the Kruskal–Wallis test with Dunn's *post-hoc* test. The correlation between two different variables was analyzed using nonparametric Spearman's correlation coefficient tests. *p* < 0.05 was considered significant in the study. To investigate the role of ABA in predicting COPD disease progression, we fitted a multiple multinomial logistic regression using R's package “nnet,” considering that while the stages are ordered the control group's order does not necessarily lie under COPD stage I. To adjust for the potential confounding role of the sample characteristics on the predictive value of ABA, we included demographic variables and immune-regulatory factors and inflammatory markers to the model selection process by fitting individual factors and select significant ones. The best model was selected based on the remained significant variables in a multiple regression model. Since our main purpose is to explore the role of ABA, the term ABA is always included in the model selection process instead of letting the automatic algorithm to decide on whether or not ABA is to be remained in the final model. Due to a wide variety in the range of analyte measurements, all the continuous covariates were scaled and centered before fitting the model and the coefficient was transformed back to interpret on the original data scale. The software used for most of the graphical representations was GraphPad Prism version 5.0, whereas statistical modeling was performed using R version 4.0 and the multinomial regression was fitted using package “nnet.”

## Results

### Decreased ABA Levels in Peripheral Blood of COPD Patients With the Lowest Levels Observed in Settings of Active Lung Inflammation

**Figure 1:**
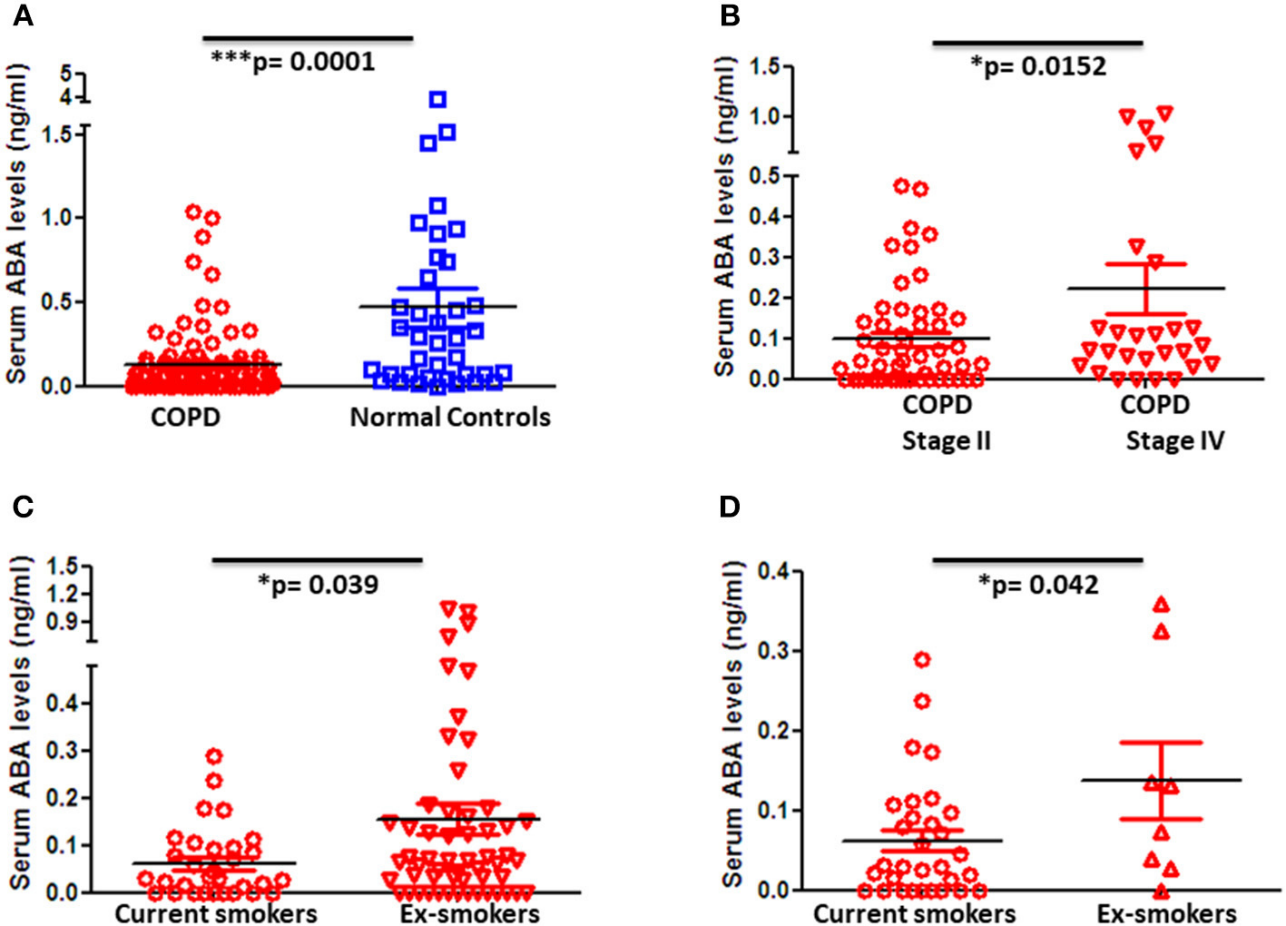
Blood levels of ABA in patients with COPD and controls and subcategories of COPD based on disease severity and smoking habits. Circulating serum levels of ABA in **(A)** COPD patients (*n* = 98) and age- and gender-matched controls (*n* = 38), **(B)** GOLD classified moderate (Stage II) (*n* = 52) and severe (Stage IV) (*n* = 28) COPD patients, COPD patients with current smoking habits, **(C)** ex-smokers, and **(D)** and those who never smoked. Individual dots represent data obtained from individual subjects. Statistical analysis was done by either nonparametric Mann–Whitney test or unpaired Student *t*-test.

Blood samples of 101 COPD patients and 57 age- and gender-matched controls were collected, and ABA levels in sera were determined by LC-MS/MS. Strikingly, ABA serum levels were significantly decreased in COPD patients when compared to control donors (Figure 1A). Furthermore, when COPD patients were stratified based on their disease stage according to the GOLD classification, we found that stage IV COPD patients who are considered end-stage with extensive tissue remodeling and emphysema but at the same time with low-grade active inflammation tend to exhibit higher ABA serum levels compared to stage II COPD patients with mild to moderate disease severity, who are still with active and progressive inflammation (Figure 1B). Further classification of COPD patients based on their cigarette smoking history revealed that serum ABA levels are lowest in current smokers compared to those patients who either quitted smoking (Figure 1C) or were nonsmokers (Figure 1D). Further quantification of ABA levels in the BALF of COPD patients and controls exhibited below detection limits.

Since COPD is considered to be an age-associated disease, we further classified COPD patients with respect to their age. Interestingly, ABA levels increase with age and this effect is statistically significant in COPD patients but not in control subjects (Supplementary Figures 1A–C). Of note, ABA serum levels positively correlate with the age of stage IV COPD patients (Supplementary Figure 1D).

### Increased Expression of PPAR-γ on Peripheral Blood Mononuclear Cells of COPD Patients

**Figure 2:**
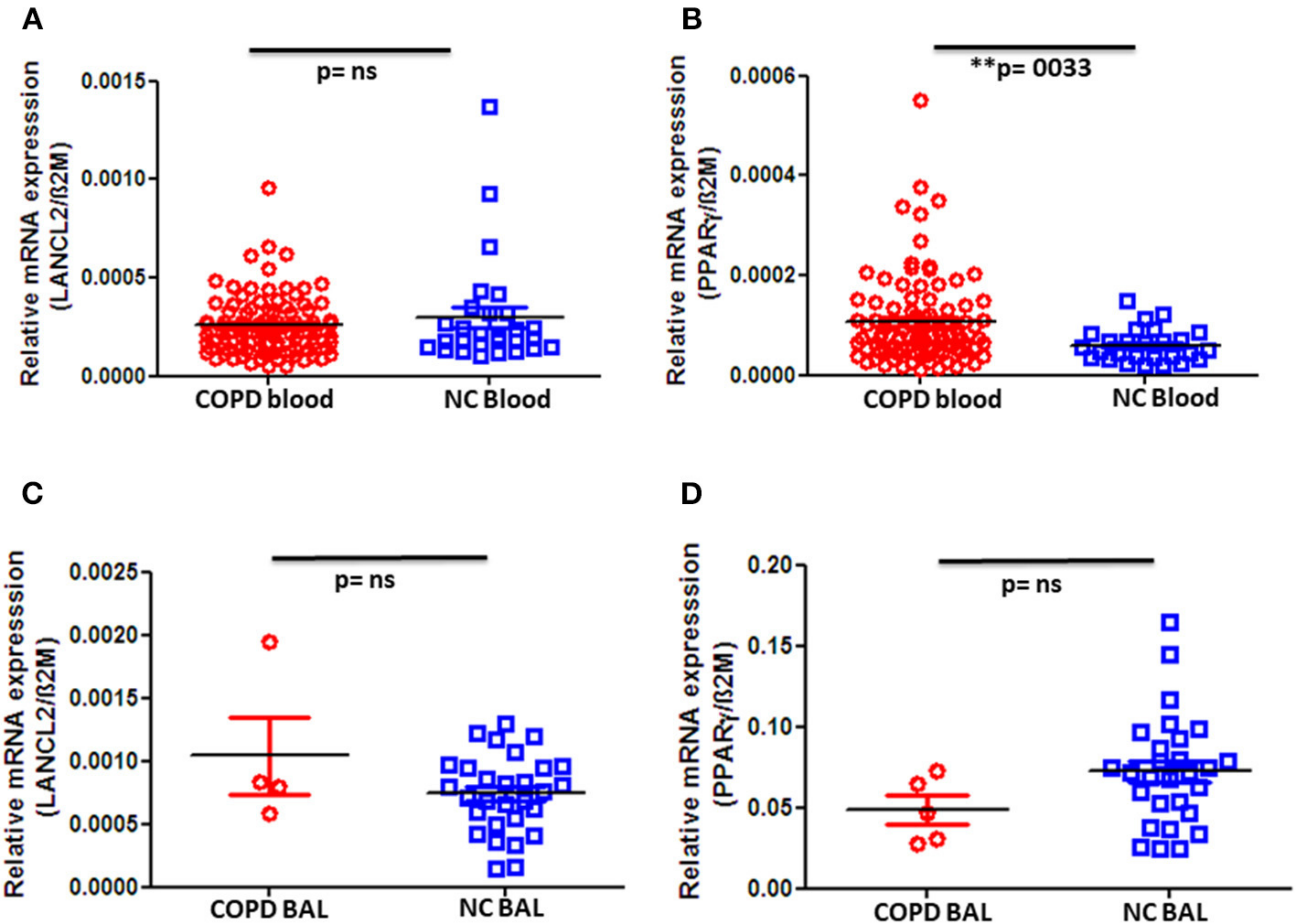
Gene expression levels of LANCL2 and PPAR-γ in patients with COPD and controls. Gene expression levels of **(A)** LANCL2 and **(B)** PPAR-γ on peripheral blood cells from COPD patients (*n* = 94) and age- and gender-matched controls (*n* = 28 and 27, respectively). Bronchioalveolar lavage cell gene expression of **(C)** LANCL2 (*n* = 4) and **(D)** PPAR-γ (*n* = 5) in COPD patients and control subjects (*n* = 29 and 28, respectively). The individual dots represent data obtained from individual subjects. Statistical analysis was done by either nonparametric Mann–Whitney test or unpaired Student *t*-test.

Next, we quantified the gene expression of LANCL2 and PPAR-γ in PBMC as well as BAL cells. Although we did not observe any significant difference in LANCL2 expression in peripheral blood cells between COPD patients and control groups (Figure 2A), we indeed found a significant increase in PPAR-γ expression on PBMC from COPD patients when compared to normal controls (Figure 2B). In particular, advanced (stage IV) COPD exhibited significantly higher expression levels of PPAR-γ when compared to the control subjects (Supplementary Figure 2C). In contrast, no changes in LANCL2 and PPAR-γ expression were observed on BAL cells from COPD patients compared to control subjects (Figures 2C,D). Furthermore, we did not observe significant differences in PBMC expression levels of LANCL2 and PPAR-γ among COPD patients, either when classified regarding their disease severity (GOLD stage I–IV) or when categorized based on their smoking habits (data not shown). In conclusion, decreased ABA levels in the circulation of COPD patients are accompanied by increased expression of PPAR-γ on PBMC.

### Decreased ABA Serum Levels and Altered PPAR-γ and LANCL2 Expression on Peripheral Blood Mononuclear Cells in Asthmatic Patients

**Figure 3:**
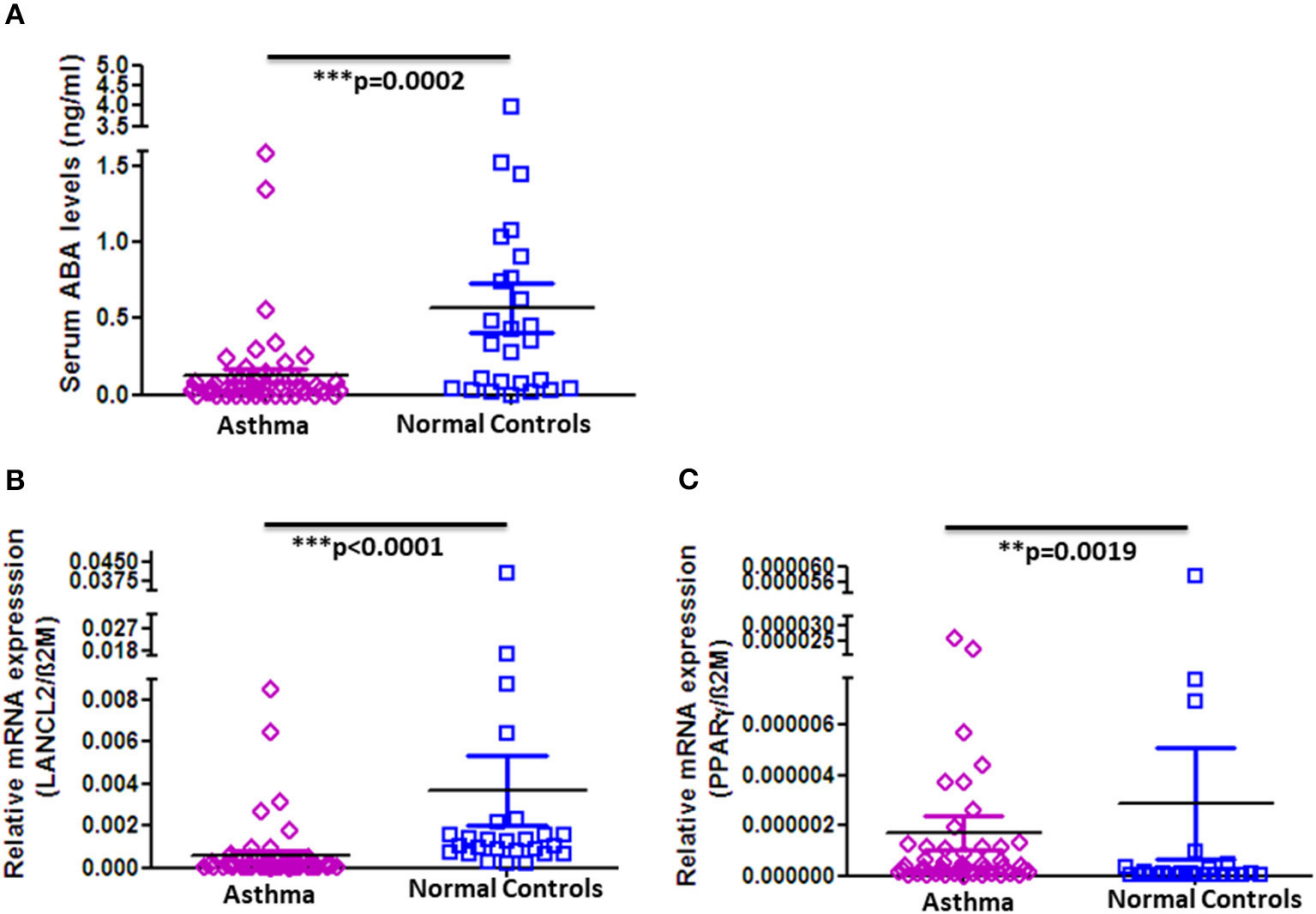
ABA serum levels and LANCL2 and PPAR-γ gene expression on blood cells in asthma patients and controls. **(A)** Circulating serum levels of ABA in asthma patients (*n* = 52) and age- and gender-matched controls (*n* = 26). Blood cell gene expression of **(B)** LANCL2 and **(C)** PPAR-γ in asthma patients (*n* = 52) and age- and gender-matched controls (*n* = 26). The individual dots represent data obtained from individual subjects. Statistical analysis was done by either nonparametric Mann–Whitney test or unpaired Student *t*-test.

In order to determine whether the observed decrease in ABA serum levels and altered expression of PPAR-γ and LANCL2 on PBMC are unique for COPD or would also apply for other chronic respiratory diseases, we analyzed the serum concentration of ABA in asthma patients and age- and gender-matched normal controls. Strikingly, as in COPD patients, ABA serum levels are indeed significantly reduced in asthmatics when compared to normal controls (Figure 3A). However, there are no differences with respect to higher or lower ABA levels distinguishing the stages of asthma progression (Supplementary Figure 7). Interestingly, we observed significantly reduced expressions of LancL2 and PPAR-γ on PBMC from asthma patients compared to the normal controls (Figures 3B,C). In conclusion, the reduced ABA concentration in the circulation of COPD patients and asthmatics was observed, whereas the PBMC expressions of PPAR-γ and LANCL2 were altered.

### ABA Serum Levels Positively Correlate With Immune-Regulatory Factors While Inversely Correlating With Inflammatory Markers in COPD Patients

**Figure 4:**
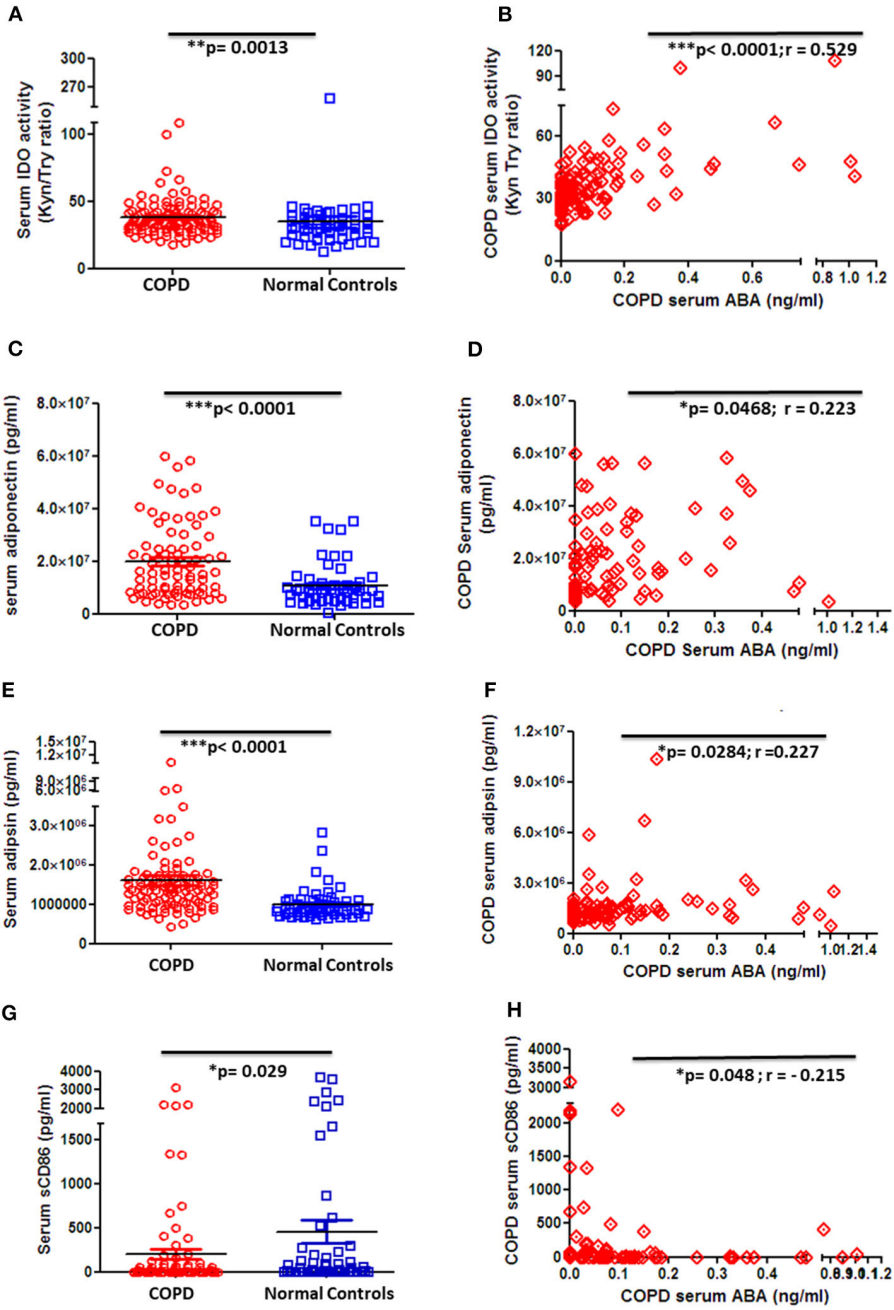
Circulating serum levels of anti-inflammatory, insulin-sensitizing, and inflammatory markers in patients with COPD and controls. **(A)** Serum activity of immune-regulatory enzyme, indoleamine-2,3-dioxygenase (IDO), by measuring the kynurenine-to-tryptophan ratio in COPD patients (*n* = 101) and controls (*n* = 54) and **(B)** correlation of serum ABA levels and IDO activity among COPD patients (*n* = 97). Serum levels of **(C)** the anti-inflammatory adipocytokine, adiponectin, in COPD patients and controls and **(D)** correlation of serum ABA levels and adiponectin among COPD patients. Serum levels of **(E)** insulin-sensitizing adipsin in COPD patients and controls and **(F)** correlation of serum ABA levels and adipsin among COPD patients. Serum levels of **(G)** the proinflammatory costimulatory molecule sCD86 in COPD patients and controls and **(H)** correlation of serum ABA levels and sCD86 among COPD patients. The individual dots represent data obtained for individual subject. Statistical analysis was done by either nonparametric Mann–Whitney test or unpaired Student *t*-test. Correlation test was performed using nonparametric Spearman's rank correlation coefficient test.

In order to gain knowledge on the potential role of ABA in COPD, we next thought to correlate ABA serum levels with clinically relevant markers that are associated with disease severity and COPD-related comorbidities. Quantification of the immune-regulatory enzyme IDO, the anti-inflammatory adipocytokine adiponectin, and the insulin-sensitizing adipocytokine adipsin revealed that the levels of all factors are significantly elevated in COPD patient sera when compared to controls (Figures 4A,C,E). Of note, although ABA serum concentration is generally decreased in COPD patients, it exhibits a significant and positive correlation to the anti-inflammatory analytes IDO (Figure 4B), adiponectin (Figure 4D), and insulin-sensitizing adipsin (Figure 4F). Furthermore, serum levels of the inflammation marker soluble(s) CD86 are significantly decreased among COPD patients when compared to controls (Figure 4G) and intriguingly negatively correlate with ABA serum levels (Figure 4H). We have also measured other COPD-related markers including CRP, resistin, MMP-9, leptin, and pro-inflammatory cytokines (IL-6, IL-1β, TNF-α, and IL-8). While serum cytokines were mostly below the level of detection (data not shown), we observed increased CRP levels and decreased resistin and MMP-9 levels in the circulation of COPD patients when compared to controls (Supplementary Figures 6A–C). However, we did not observe a significant correlation between these factors and the levels of ABA (data not shown). Together, correlation studies revealed that ABA serum levels in COPD patients are associated with immune-regulatory mediators and thereby follow a similar pattern as observed for other disease-associated immune-regulatory mediators.

### ABA as a New Possible Biomarker for Disease Stages in COPD

To select the best predictive model for COPD disease progression based on ABA, we have included sex, age, immune-regulatory factors, and inflammatory markers as covariates in model selection. The best model was selected based on the individual variables' statistical significance, and those that remained statistically significant in a multiple regression model include ABA, CRP, and adipsin, adjusting for individual age—which was also found to be significant (Table 3). The model shows that ABA is mostly negatively associated with COPD stages; for example, with every unit increase in ABA, the log odds of being in stage I compared to having no COPD reduced by 2.98, or in other words, the relative risk of being in stage I compared to having no COPD reduced by exp (−2.98) ~ 5% for every unit increase in ABA level. However, the effect is no longer as clear in COPD stages III and IV. The model also confirms the role of CRP and adipsin in predicting COPD disease state.

**Table 3:** Multinomial logistic regression model.

**No COPD**	**Stage**	**Coefficient**	**SE**	***p*-value**	**[95% Confidence interval]**
		**(base outcome)**			
I	ABA	−2.98	1.36	0.03	−5.65 to −0.31
	age	0.07	0.02	<0.001	0.03 to 0.12
	CRP	20.25	4.98	0.001	10.48 to 30.01
	Adipsin	5.23	2.23	0.02	0.87 to 9.59
II	ABA	−2.53	0.8	<0.001	−4.11 to −0.96
	age	0.1	0.02	<0.001	0.06 to 0.14
	CRP	20.01	4.98	<0.001	10.25 to 29.77
	Adipsin	5.36	2.21	0.02	1.03 to 9.68
III	ABA	−2.68	1.58	0.09	−5.77 to 0.42
	age	0.07	0.02	0.01	0.02 to 0.12
	CRP	18.97	5.35	<0.001	8.49 to 29.44
	Adipsin	5.13	2.26	0.02	0.71 to 9.56
IV	ABA	−1.3	0.72	0.07	−2.72 to 0.11
	age	0.09	0.02	<0.001	0.05 to 0.14
	CRP	19.95	4.98	<0.001	10.18 to 29.72
	Adipsin	5.14	2.21	0.02	0.81 to 9.46

*All the coefficients are compared relatively to no COPD group and dependent on the raw scale of the variable (n = 126)*.

**Figure 5:**
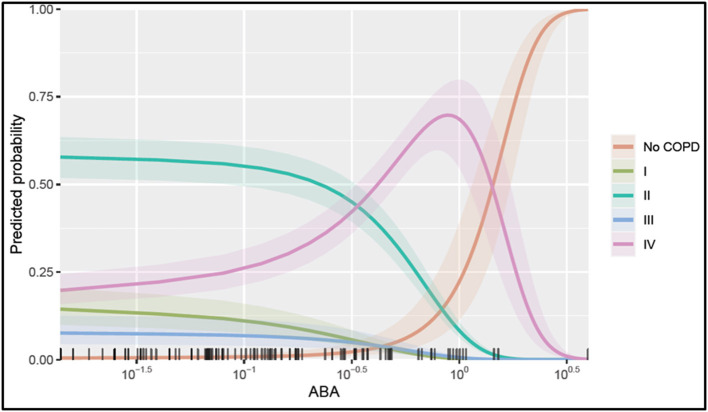
Probability of being in one of the COPD stages over serum ABA levels. The plotted values were calculated assuming an average individual in the sample that is conditional on the mean value of the other covariates (condition in a patient aged 65, a CRP value at 10^0.83^, and adipsin value at 10^6.27^) and their 95% confidence interval of the prediction. The rug at the plot's bottom margin represents the data frequency used in the analysis. The statistical modeling was performed using R 4.0.

To illustrate the marginal effect of ABA in a multivariable regression model presented in Table 3, we conditioned on the mean value of the model covariates including CRP, age, and adipsin and plotted (Figure 5). It shows the predicted probability of being in one of the COPD stages holding the rest of the covariates at their average value, noting that in case different values were chosen, the magnitude plotted will be different but the shape of the effect will remain the same as it is in the case of the multivariable regression model. Figure 5 shows that serum ABA levels lower than 10^−5^ may correspond to COPD stage II, while ABA levels between 10^−5^ and 10^0^ could correspond more likely to COPD stage IV. For values of ABA over 10^0.3^, the probability of not presenting COPD is largely increased. Note in Figure 5 that the confidence intervals for the probabilities of COPD I and III are overlapping; thus, serum ABA levels would not help to distinguish between these stages of COPD.

In summary, quantification of ABA serum levels in a large patient cohort revealed that COPD is associated with reduced concentrations of this factor in the circulation. Moreover, the lowest ABA concentrations were associated with conditions of active lung inflammation.

## Discussion

Supplementation of ABA by feeding an ABA-rich diet has been previously shown in animal models for chronic inflammatory disorders to ameliorate inflammatory processes (47, 48, 50, 51). In the present study, we show for the first time that baseline levels of ABA in the circulation are significantly reduced in COPD patients and asthmatics, which is consistent with the clinical findings in patients suffering from type 2 diabetes and systemic sclerosis (58, 59). Reduced ABA concentration might either be the consequence of intrinsic defects in the cellular ABA synthesis or, alternatively, due to the enhanced ABA consumption upon binding to its receptor LANCL2. Hence, in our study, it might be possible that LANCL2-bound ABA might have an induced elevated expression of PPAR-γ in PBMC of COPD patients. This very much resembles the concept of the agonistic action of pioglitazone (an FDA-approved synthetic PPAR-γ agonist and antidiabetic drug) to activate PPAR-γ which was already proven to be effective in controlling inflammatory responses in other chronic inflammatory diseases such as multiple sclerosis and systemic lupus erythematosus (60, 61). Most of the COPD patients enrolled in our study are treated with β-agonist, muscarinic antagonist, inhaled corticosteroids (ICS), or a combination of all. A previous report showed that the expression of PPAR-γ in sputum cells from COPD patients was promoted by the long-acting β2 agonist formoterol (62). Interestingly, in our study we noticed that the expression of PPAR-γ was generally lower in PBMC from women, irrespective of their health status (Supplementary Figure 4). This implies that the overall level of PPAR-γ expression could be gender-specific and that the observed low PPAR-γ expression level in women might increase the likelihood for a faster disease progression in women than in men. This hypothesis is corroborated by the findings of accelerated decline in lung function, greater risk of airway obstruction, and physical limitation, which are prevalent and more frequent in aged COPD women when compared to COPD men (63, 64). A sharp drop in the estrogen levels could further explain the notion of reduced PPAR-γ expression among elderly women (65). Although the serum concentration of ABA did not differ between genders (Supplementary Figure 3), we may speculate that the elevated level of PPAR-γ expression on PBMC might reflect ABA-mediated effects in modulating and alleviating the disease activity, as it is evident that the loss of PPAR-γ impaired the anti-inflammatory attributes of ABA (49). Since the expression of LANCL2 and PPAR-γ in asthmatics differs from that of COPD patients, the ABA-related outcome might be different in asthmatics (Supplementary Figures 3B,C, 7C), which requires further investigations.

Understanding the reason behind the increase in ABA serum levels with advancement of COPD remains an interesting but challenging issue. It is well-endorsed that at the beginning COPD is marked by chronic airway inflammation that usually progresses to emphysema in advanced stages, a condition that is characterized by irreversible bronchial narrowing and alveolar hyperinflation (5). Increased ABA levels in the advanced stage of COPD might be associated with the so-called inflammaging where low-grade inflammation is expected to occur during aging (66). To support this, our study shows a strong correlation between ABA concentration in the circulation and the age of the entire COPD (Supplementary Figures 1A,C) as well as asthma (Supplementary Figure 7A) cohort. Furthermore, ABA levels increase from stage I to IV COPD patients (Supplementary Figure 2A). Of note, most patients with progressive COPD are above 65 years old, further supporting the notion that ABA levels increase with decreasing inflammation. One of the major characteristics of low-grade inflammation during aging is that the immune system undergoes age-associated alterations leading to immune senescence along with a decline in immune functions (67, 68). We may therefore speculate that there is a link between aging-related disease processes in progressing COPD and immune senescence which might result in the observed increase in ABA levels both in advanced-stage COPD patients and in the elderly. This is supported by a report dealing with plant physiology that described ABA to promote senescence in leaves to be associated with aging in plants (69). Due to conserved functions of ABA both in plants and in mammals, we consider an analogous trait to take place in humans. In order to gain additional information regarding potential alterations of ABA levels at the site of inflammation, i.e., the lung, we also measured ABA levels in BALF from both COPD patients and control subjects. However, irrespective of whether or not the donor suffered from COPD and in contrast to serum, ABA levels in BALF were below the level of detection (data not shown).

We noticed an increase in serum IDO activity in our COPD cohort (Figure 4), which might be stimulated by IFN-γ or other pro-inflammatory cytokines (70) that are released in consequence of ongoing lung inflammation (71). IDO might suppress inflammatory responses during chronic inflammation (72) in COPD patients, which however needs further investigation. IDO is a first-step, rate-limiting enzyme produced during tryptophan catabolism, which acts as an immune-regulatory metabolite (73), indicating its significance during acute exacerbation as well as stable state in COPD, respectively. In fact, the combination of corticosteroids and β2 agonists possesses the potential to enhance IDO activity (74) and thus could in part explain increased IDO levels in COPD patients that apply these medications on a regular basis. Next to IDO, our analyses revealed elevated levels of the serum adipocytokine adiponectin in COPD patients (Figure 4). Increased adiponectin level might be due to the treatment to COPD patients with β agonists (75), and we consider adiponectin to display anti-inflammatory attributes by inhibiting the expression of the pro-inflammatory cytokine TNFα and the transcription factor nuclear factor kappa beta (NF-κb) (76) and by mediating lung-protective properties (75). Of note, adiponectin levels were also shown to be increased upon treatment with the FDA-approved PPAR-γ agonist thiazolidinediones (77), and in our study we indeed find an increased PPAR-γ expression on PBMC from COPD patients. Although another study has demonstrated that plasma levels of adipokines, and here mainly leptin and not adiponectin, could be indicative for COPD emphysema and severity (33), we could not demonstrate a role of leptin in our COPD cohort and thereby could not compare it as a control biomarker for ABA in our study. Besides, increased levels of sCD86 in the circulation were shown to be tightly linked to the immunopathogenesis of several chronic inflammatory diseases, such as systemic lupus erythematosus, rheumatoid arthritis, abdominal aortic aneurysm, and acute asthma (78–81). In direct contrast to this, the COPD cohort studied here exhibits reduced serum levels of sCD86. This might be due to the effect of β-2-agonists (β2A), as it was reported that β2A receptor stimulation decreases the expression of surface-bound CD86 on dendritic cells (82). We may thus speculate that regular medications in COPD patients result in decreased sCD86 serum levels either due to reduced shedding of the CD86 from the cell membrane or due to the reduced expression of surface-bound CD86 on antigen-presenting cells. Taken together, the ABA level in the circulation directly correlates with the levels of the immune-regulatory and anti-inflammatory markers IDO and adiponectin while inversely correlating with the serum level of the pro-inflammatory marker sCD86, suggesting an overall anti-inflammatory function of ABA in COPD.

Compelling evidence exists regarding the significance of ABA in glucose homeostasis by increasing insulin sensitivity of and improving glucose uptake by cells (41, 51, 83). In line with this, we demonstrate elevated levels of the adipocytokine adipsin in the circulation of COPD patients. The insulin-sensitizing adipsin (84) is expressed by adipocytes, and its expression was shown to be dysregulated in models of obesity and diabetes. Here, its mode of action was demonstrated not only in improving insulin sensitivity but also in enhancing the insulin secretion by pancreatic β-cells, in contrast to adiponectin which is well-known to enhance insulin sensitivity but lacks the capacity to stimulate pancreatic β cells to release insulin (85–87). In our study, increased adipsin levels could relate to a relatively low number of COPD patients enrolled in the survey with comorbidities for metabolic conditions, including diabetes and hypercholesterolemia. Remarkably, our study defines a direct relation between ABA and the diabetes protective factor, adipsin. Future studies are needed to further clarify the diagnostic value of ABA and adipsin levels to stratify COPD patients according to their potential comorbidities. Interestingly, ABA serum levels negatively correlate with BMI, i.e., they decrease with increasing body weight in the COPD cohort (Supplementary Figure 5). Thus, ABA might be considered as a surrogate-circulating factor linking adipocytes and obesity to β cell function among COPD patients with the respective comorbidities, which requires further specific investigations.

## Conclusion

Taken together, we conclude that ABA serum levels are positively associated with immune-regulatory mediators and advanced stage of COPD. Moreover, both conventional statistical analysis and application of the multinomial logistic regression model revealed that ABA serum levels can be regarded as a potential biomarker in distinguishing early and late stages of COPD.

## Data Availability Statement

The raw data supporting the conclusions of this article will be made available by the authors, without undue reservation.

## Ethics Statement

The studies involving human participants were reviewed and approved by Prof. Dr. med. Christof Huth, Ethic Commission, Medical Faculty, Otto-von-Guericke University Hospital, Magdeburg, Germany. The patients/participants provided their written informed consent to participate in this study.

## Author Contributions

PV contributed to the conception and design of the study, fund raising, performed experiments and statistical analysis, and wrote the first draft of the manuscript. DB contributed to the conception and design of the study, fund raising, and wrote the section of the manuscript. JS contributed to the fund raising and wrote the section of the manuscript. JW wrote the section of the manuscript, contributed the intellectual reading, and correction of the manuscript. NW contributed to the sample collections. KB performed the experiments. EH-V and VN contributed to the statistical analysis and wrote the section of the manuscript. DF performed the experiments and wrote the section of the manuscript. HO contributed to the conception, performed the experiments, and wrote the section of the manuscript. QH contributed to the sample collections, performed the experiments, and wrote the section of the manuscript. All the authors have read and approved the submission of the manuscript.

## Conflict of Interest

The authors declare that the research was conducted in the absence of any commercial or financial relationships that could be construed as a potential conflict of interest.

## References

[1] Soriano JB Abajobir AA Abate KH Abera SF Agrawal A Ahmed MB. Global, regional, and national deaths, prevalence, disability-adjusted life years, and years lived with disability for chronic obstructive pulmonary disease and asthma, 1990-2015: a systematic analysis for the Global Burden of Disease Study 2015. Lancet Respir Med. (2017) 5:691–706. 10.1016/S2213-2600(17)30293-X 28822787 PMC5573769

[2] Wisnivesky J De-Torres JP. The global burden of pulmonary diseases: most prevalent problems and opportunities for improvement. Ann Glob Health. (2019) 85:1. 10.5334/aogh.2411 30741502 PMC7052315

[3] Soriano JB Kendrick PJ Paulson KR Gupta V Abrams EM Adedoyin RA. Prevalence and attributable health burden of chronic respiratory diseases, 1990-2017: a systematic analysis for the Global Burden of Disease Study 2017. Lancet Respir Med. (2020) 8:585–96. 10.1016/S2213-2600(20)30105-3 32526187 PMC7284317

[4] Hogg JC Macklem PT Thurlbeck WM. Site and nature of airway obstruction in chronic obstructive lung disease. N Engl J Med. (1968) 278:1355–60. 10.1056/NEJM196806202782501 5650164

[5] McDonough JE Yuan R Suzuki M Seyednejad N Elliott WM Sanchez PG. Small-airway obstruction and emphysema in chronic obstructive pulmonary disease. N Engl J Med. (2011) 365:1567–75. 10.1056/NEJMoa1106955 22029978 PMC3238466

[6] Zanini A Chetta A Saetta M Baraldo S Castagnetti C Nicolini G. Bronchial vascular remodelling in patients with COPD and its relationship with inhaled steroid treatment. Thorax. (2009) 64:1019–24. 10.1136/thx.2009.114629 19736178

[7] Allinson JP Hardy R Donaldson GC Shaheen SO Kuh D Wedzicha JA. The presence of chronic mucus hypersecretion across adult life in relation to chronic obstructive pulmonary disease development. Am J Respir Crit Care Med. (2016) 193:662–72. 10.1164/rccm.201511-2210OC 26695373 PMC4824943

[8] Tuder RM Yoshida T Arap W Pasqualini R Petrache I. State of the art. Cellular and molecular mechanisms of alveolar destruction in emphysema: an evolutionary perspective. Proc Am Thorac Soc. (2006) 3:503–10. 10.1513/pats.200603-054MS 16921129 PMC2647641

[9] Konietzke P Wielpütz MO Wagner WL Wuennemann F Kauczor HU Heussel CP. Quantitative CT detects progression in COPD patients with severe emphysema in a 3-month interval. Eur Radiol. (2020) 30:2502–12. 10.1007/s00330-019-06577-y 31965260

[10] Papi A Brightling C Pedersen SE Reddel HK. Asthma. Lancet. (2018) 391:783–800. 10.1016/S0140-6736(17)33311-1 29273246

[11] Maselli DJ Hardin M Christenson SA Hanania NA Hersh CP Adams SG. Clinical approach to the therapy of asthma-COPD overlap. Chest. (2019) 155:168–77. 10.1016/j.chest.2018.07.028 30077690 PMC6688980

[12] Anthonisen NR Connett JE Kiley JP Altose MD Bailey WC Buist AS. Effects of smoking intervention and the use of an inhaled anticholinergic bronchodilator on the rate of decline of FEV1. The Lung Health Study. JAMA. (1994) 272:1497–505. 10.1001/jama.1994.03520190043033 7966841

[13] Wilson D Adams R Appleton S Ruffin R. Difficulties identifying and targeting COPD and population-attributable risk of smoking for COPD: a population study. Chest. (2005) 128:2035–42. 10.1378/chest.128.4.2035 16236852

[14] Krzyzanowski M Jedrychowski W Wysocki M. Factors associated with the change in ventilatory function and the development of chronic obstructive pulmonary disease in a 13-year follow-up of the Cracow Study. Risk of chronic obstructive pulmonary disease. Am Rev Respir Dis. (1986) 134:1011–9. 10.1164/arrd.1986.134.5.1011 3777663

[15] Platts-Mills TA Tovey ER Mitchell EB Moszoro H Nock P Wilkins SR. Reduction of bronchial hyperreactivity during prolonged allergen avoidance. Lancet. (1982) 2:675–8. 10.1016/S0140-6736(82)90709-7 6126624

[16] Del Giacco SR Bakirtas A Bel E Custovic A Diamant Z Hamelmann E. Allergy in severe asthma. Allergy. (2017) 72:207–20. 10.1111/all.13072 27775836

[17] Fried TR Vaz Fragoso CA Rabow MW. Caring for the older person with chronic obstructive pulmonary disease. JAMA. (2012) 308:1254–63. 10.1001/jama.2012.12422 23011715 PMC3815613

[18] Mercado N Ito K Barnes PJ. Accelerated ageing of the lung in COPD: new concepts. Thorax. (2015) 70:482–9. 10.1136/thoraxjnl-2014-206084 25739910

[19] Trivedi M Denton E. Asthma in children and adults-what are the differences and what can they tell us about asthma? Front Pediatr. (2019) 7:256. 10.3389/fped.2019.00256 31294006 PMC6603154

[20] Marçôa R Rodrigues DM Dias M Ladeira I Vaz AP Lima R. Classification of Chronic Obstructive Pulmonary Disease (COPD) according to the new Global Initiative for Chronic Obstructive Lung Disease (GOLD) 2017: Comparison with GOLD (2011). Copd. (2018) 15:21–6. 10.1080/15412555.2017.1394285 29161163

[21] Buhl R Bals R Baur X Berdel D Criée C-P Gappa M. [Guideline for the Diagnosis and Treatment of Asthma - Guideline of the German Respiratory Society and the German Atemwegsliga in Cooperation with the Paediatric Respiratory Society and the Austrian Society of Pneumology]. Pneumologie. (2017) 71:849–919. 10.1055/s-0043-119504 29216678

[22] Padem N Saltoun C. Classification of asthma. Allergy Asthma Proc. (2019) 40:385–8. 10.2500/aap.2019.40.4253 31690376

[23] Arteaga-Badillo DA Portillo-Reyes J Vargas-Mendoza N Morales-González JA Izquierdo-Vega JA Sánchez-Gutiérrez M. Asthma: new integrative treatment strategies for the next decades. Medicina (Kaunas). (2020) 56:438. 10.3390/medicina56090438 32872366 PMC7558718

[24] Mauer Y Taliercio RM. Managing adult asthma: the 2019 GINA guidelines. Cleve Clin J Med. (2020) 87:569–75. 10.3949/ccjm.87a.19136 32868307

[25] Boulet LP Reddel HK Bateman E Pedersen S FitzGerald JM O'Byrne PM. The global initiative for asthma (GINA): 25 years later. Eur Respir J. (2019) 54:1900598. 10.1183/13993003.00598-2019 31273040

[26] Brusselle GG Joos GF Bracke KR. New insights into the immunology of chronic obstructive pulmonary disease. Lancet. (2011) 378:1015–26. 10.1016/S0140-6736(11)60988-4 21907865

[27] Holloway RA Donnelly LE. Immunopathogenesis of chronic obstructive pulmonary disease. Curr Opin Pulm Med. (2013) 19:95–102. 10.1097/MCP.0b013e32835cfff5 23325031

[28] Nuñez A Marras V Harlander M Mekov E Esquinas C Turel M. Association between routine blood biomarkers and clinical phenotypes and exacerbations in chronic obstructive pulmonary disease. Int J Chron Obstruct Pulmon Dis. (2020) 15:681–90. 10.2147/COPD.S240720 32280207 PMC7127861

[29] Maneechotesuwan K Kasetsinsombat K Wongkajornsilp A Barnes PJ. Decreased indoleamine 2,3-dioxygenase activity and IL-10/IL-17A ratio in patients with COPD. Thorax. (2013) 68:330–7. 10.1136/thoraxjnl-2012-202127 23255616

[30] Fermont JM Masconi KL Jensen MT Ferrari R Di Lorenzo VAP Marott JM. Biomarkers and clinical outcomes in COPD: a systematic review and meta-analysis. Thorax. (2019) 74:439–46. 10.1136/thoraxjnl-2018-211855 30617161 PMC6484697

[31] Skyba P Ukropec J Pobeha P Ukropcova B Joppa P Kurdiova T. Metabolic phenotype and adipose tissue inflammation in patients with chronic obstructive pulmonary disease. Mediators Inflamm. (2010) 2010:173498. 10.1155/2010/173498 21197447 PMC3010654

[32] Franssen FM O'Donnell DE Goossens GH Blaak EE Schols AM. Obesity and the lung: 5. Obesity COPD. Thorax. (2008) 63:1110–7. 10.1136/thx.2007.086827 19020276

[33] Oh YM Jeong BH Woo SY Kim SY Kim H Lee JH. Association of plasma adipokines with chronic obstructive pulmonary disease severity and progression. Ann Am Thorac Soc. (2015) 12:1005–12. 10.1513/AnnalsATS.201501-005OC 26010877

[34] Moon SW Leem AY Kim YS Lee JH Kim TH Oh YM. Low serum lymphocyte level is associated with poor exercise capacity and quality of life in chronic obstructive pulmonary disease. Sci Rep. (2020) 10:11700. 10.1038/s41598-020-68670-3 32678181 PMC7366616

[35] Schroeder JI Kuhn JM. Plant biology: abscisic acid in bloom. Nature. (2006) 439:277–8. 10.1038/439277a 16421556

[36] Le Page-Degivry MT Bidard JN Rouvier E Bulard C Lazdunski M. Presence of abscisic acid, a phytohormone, in the mammalian brain. Proc Natl Acad Sci U S A. (1986) 83:1155–8. 10.1073/pnas.83.4.1155 2937056 PMC323030

[37] Rabbani MA Maruyama K Abe H Khan MA Katsura K Ito Y. Monitoring expression profiles of rice genes under cold, drought, and high-salinity stresses and abscisic acid application using cDNA microarray and RNA gel-blot analyses. Plant Physiol. (2003) 133:1755–67. 10.1104/pp.103.025742 14645724 PMC300730

[38] Anderson JP Badruzsaufari E Schenk PM Manners JM Desmond OJ Ehlert C. Antagonistic interaction between abscisic acid and jasmonate-ethylene signaling pathways modulates defense gene expression and disease resistance in Arabidopsis. Plant Cell. (2004) 16:3460–79. 10.1105/tpc.104.025833 15548743 PMC535886

[39] Bruzzone S Moreschi I Usai C Guida L Damonte G Salis A. Abscisic acid is an endogenous cytokine in human granulocytes with cyclic ADP-ribose as second messenger. Proc Natl Acad Sci U S A. (2007) 104:5759–64. 10.1073/pnas.0609379104 17389374 PMC1832220

[40] Magnone M Bruzzone S Guida L Damonte G Millo E Scarfì S. Abscisic acid released by human monocytes activates monocytes and vascular smooth muscle cell responses involved in atherogenesis. J Biol Chem. (2009) 284:17808–18. 10.1074/jbc.M809546200 19332545 PMC2719419

[41] Bruzzone S Ameri P Briatore L Mannino E Basile G Andraghetti G. The plant hormone abscisic acid increases in human plasma after hyperglycemia and stimulates glucose consumption by adipocytes and myoblasts. Faseb J. (2012) 26:1251–60. 10.1096/fj.11-190140 22075645

[42] Sturla L Fresia C Guida L Grozio A Vigliarolo T Mannino E. Binding of abscisic acid to human LANCL2. Biochem Biophys Res Commun. (2011) 415:390–5. 10.1016/j.bbrc.2011.10.079 22037458

[43] Sturla L Fresia C Guida L Bruzzone S Scarfì S Usai C. LANCL2 is necessary for abscisic acid binding and signaling in human granulocytes and in rat insulinoma cells. J Biol Chem. (2009) 284:28045–57. 10.1074/jbc.M109.035329 19667068 PMC2788856

[44] Bassaganya-Riera J Guri AJ Lu P Climent M Carbo A Sobral BW. Abscisic acid regulates inflammation via ligand-binding domain-independent activation of peroxisome proliferator-activated receptor gamma. J Biol Chem. (2011) 286:2504–16. 10.1074/jbc.M110.160077 21088297 PMC3024745

[45] Heming M Gran S Jauch SL Fischer-Riepe L Russo A Klotz L. Peroxisome proliferator-activated receptor-γ modulates the response of macrophages to lipopolysaccharide and glucocorticoids. Front Immunol. (2018) 9:893. 10.3389/fimmu.2018.00893 29867927 PMC5949563

[46] Park YS Lillehoj EP Kato K Park CS Kim KC. PPARγ inhibits airway epithelial cell inflammatory response through a MUC1-dependent mechanism. Am J Physiol Lung Cell Mol Physiol. (2012) 302:L679–87. 10.1152/ajplung.00360.2011 22268120 PMC3330763

[47] Guri AJ Hontecillas R Bassaganya-Riera J. Abscisic acid ameliorates experimental IBD by downregulating cellular adhesion molecule expression and suppressing immune cell infiltration. Clin Nutr. (2010) 29:824–31. 10.1016/j.clnu.2010.02.009 20236740 PMC2894983

[48] Hontecillas R Roberts PC Carbo A Vives C Horne WT Genis S. Dietary abscisic acid ameliorates influenza-virus-associated disease and pulmonary immunopathology through a PPARγ-dependent mechanism. J Nutr Biochem. (2013) 24:1019–27. 10.1016/j.jnutbio.2012.07.010 22995385 PMC3529771

[49] Guri AJ Hontecillas R Ferrer G Casagran O Wankhade U Noble AM. Loss of PPAR gamma in immune cells impairs the ability of abscisic acid to improve insulin sensitivity by suppressing monocyte chemoattractant protein-1 expression and macrophage infiltration into white adipose tissue. J Nutr Biochem. (2008) 19:216–28. 10.1016/j.jnutbio.2007.02.010 17618105

[50] Guri AJ Misyak SA Hontecillas R Hasty A Liu D Si H. Abscisic acid ameliorates atherosclerosis by suppressing macrophage and CD4+ T cell recruitment into the aortic wall. J Nutr Biochem. (2010) 21:1178–85. 10.1016/j.jnutbio.2009.10.003 20092994 PMC2891372

[51] Guri AJ Hontecillas R Si H Liu D Bassaganya-Riera J. Dietary abscisic acid ameliorates glucose tolerance and obesity-related inflammation in db/db mice fed high-fat diets. Clin Nutr. (2007) 26:107–16. 10.1016/j.clnu.2006.07.008 17000034

[52] Guri AJ Evans NP Hontecillas R Bassaganya-Riera J. T cell PPARγ is required for the anti-inflammatory efficacy of abscisic acid against experimental IBD. J Nutr Biochem. (2011) 22:812–9. 10.1016/j.jnutbio.2010.06.011 21109419 PMC3117068

[53] Sakthivel P Sharma N Klahn P Gereke M Bruder D. Abscisic acid: a phytohormone and mammalian cytokine as novel pharmacon with potential for future development into clinical applications. Curr Med Chem. (2016) 23:1549–70. 10.2174/0929867323666160405113129 27048335

[54] Schreiber J Langwieler S Riedel S Stein K Malfertheiner P. Occult interferon α-induced pulmonary granulomatosis despite continuation of treatment. Int J Clin Pharmacol Ther. (2014) 52:1012–6. 10.5414/CP202125 25161156

[55] Schmittgen TD Livak KJ. Analyzing real-time PCR data by the comparative C(T) method. Nat Protoc. (2008) 3:1101–8. 10.1038/nprot.2008.73 18546601

[56] Laich A Neurauter G Widner B Fuchs D. More rapid method for simultaneous measurement of tryptophan and kynurenine by HPLC. Clin Chem. (2002) 48:579–81. 10.1093/clinchem/48.3.579 11861457

[57] Widner B Werner ER Schennach H Wachter H Fuchs D. Simultaneous measurement of serum tryptophan and kynurenine by HPLC. Clin Chem. (1997) 43:2424–6. 10.1093/clinchem/43.12.2424 9439467

[58] Ameri P Bruzzone S Mannino E Sociali G Andraghetti G Salis A. Impaired increase of plasma abscisic Acid in response to oral glucose load in type 2 diabetes and in gestational diabetes. PLoS One. (2015) 10:e0115992. 10.1371/journal.pone.0115992 25723556 PMC4344322

[59] Bruzzone S Battaglia F Mannino E Parodi A Fruscione F Basile G. Abscisic acid ameliorates the systemic sclerosis fibroblast phenotype in vitro. Biochem Biophys Res Commun. (2012) 422:70–4. 10.1016/j.bbrc.2012.04.107 22560900

[60] Schmidt S Moric E Schmidt M Sastre M Feinstein DL Heneka MT. Anti-inflammatory and antiproliferative actions of PPAR-gamma agonists on T lymphocytes derived from MS patients. J Leukoc Biol. (2004) 75:478–85. 10.1189/jlb.0803402 14657213

[61] Mohammadi S Saghaeian-Jazi M Sedighi S Memarian A. Immunomodulation in systemic lupus erythematosus: induction of M2 population in monocyte-derived macrophages by pioglitazone. Lupus. (2017) 26:1318–27. 10.1177/0961203317701842 28457196

[62] Holownia A Mroz RM Noparlik J Chyczewska E Braszko JJ. Expression of CREB-binding protein and peroxisome proliferator-activated receptor gamma during formoterol or formoterol and corticosteroid therapy of chronic obstructive pulmonary disease. J Physiol Pharmacol. (2008) 59(Suppl. 6):303–9. 19218654

[63] Carter R Nicotra B Huber G. Differing effects of airway obstruction on physical work capacity and ventilation in men and women with COPD. Chest. (1994) 106:1730–9. 10.1378/chest.106.6.1730 7988192

[64] Lopez Varela MV Montes de Oca M Halbert RJ Muiño A Perez-Padilla R Tálamo C. Sex-related differences in COPD in five Latin American cities: the PLATINO study. Eur Respir J. (2010) 36:1034–41. 10.1183/09031936.00165409 20378599

[65] Park HJ Choi JM. Sex-specific regulation of immune responses by PPARs. Exp Mol Med. (2017) 49:e364. 10.1038/emm.2017.102 28775365 PMC5579504

[66] Franceschi C Bonafè M Valensin S Olivieri F De Luca M Ottaviani E. Inflamm-aging. An evolutionary perspective on immunosenescence. Ann N Y Acad Sci. (2000) 908:244–54. 10.1111/j.1749-6632.2000.tb06651.x 10911963

[67] Barnes PJ Baker J Donnelly LE. Cellular senescence as a mechanism and target in chronic lung diseases. Am J Respir Crit Care Med. (2019) 200:556–64. 10.1164/rccm.201810-1975TR 30860857

[68] Cho WK Lee CG Kim LK. COPD as a Disease of immunosenescence. Yonsei Med J. (2019) 60:407–13. 10.3349/ymj.2019.60.5.407 31016901 PMC6479124

[69] Song Y Xiang F Zhang G Miao Y Miao C Song CP. abscisic acid as an internal integrator of multiple physiological processes modulates leaf senescence onset in *Arabidopsis thaliana*. Front Plant Sci. (2016) 7:181. 10.3389/fpls.2016.00181 26925086 PMC4759271

[70] Sarkar SA Wong R Hackl SI Moua O Gill RG Wiseman A. Induction of indoleamine 2,3-dioxygenase by interferon-gamma in human islets. Diabetes. (2007) 56:72–9. 10.2337/db06-0617 17192467

[71] Schröcksnadel K Wirleitner B Winkler C Fuchs D. Monitoring tryptophan metabolism in chronic immune activation. Clin Chim Acta. (2006) 364:82–90. 10.1016/j.cca.2005.06.013 16139256

[72] Munn DH Mellor AL. Indoleamine 2,3 dioxygenase and metabolic control of immune responses. Trends Immunol. (2013) 34:137–43. 10.1016/j.it.2012.10.001 23103127 PMC3594632

[73] MacKenzie CR Heseler K Müller A Däubener W. Role of indoleamine 2,3-dioxygenase in antimicrobial defence and immuno-regulation: tryptophan depletion versus production of toxic kynurenines. Curr Drug Metab. (2007) 8:237–44. 10.2174/138920007780362518 17430112

[74] Maneechotesuwan K Supawita S Kasetsinsombat K Wongkajornsilp A Barnes PJ. Sputum indoleamine-2, 3-dioxygenase activity is increased in asthmatic airways by using inhaled corticosteroids. J Allergy Clin Immunol. (2008) 121:43–50. 10.1016/j.jaci.2007.10.011 18036645

[75] Leivo-Korpela S Lehtimäki L Vuolteenaho K Nieminen R Kööbi L Järvenpää R. Adiponectin is associated with dynamic hyperinflation and a favourable response to inhaled glucocorticoids in patients with COPD. Respir Med. (2014) 108:122–8. 10.1016/j.rmed.2013.08.016 24135487

[76] Ouchi N Kihara S Arita Y Okamoto Y Maeda K Kuriyama H. Adiponectin, an adipocyte-derived plasma protein, inhibits endothelial NF-kappaB signaling through a cAMP-dependent pathway. Circulation. (2000) 102:1296–301. 10.1161/01.CIR.102.11.1296 10982546

[77] Sulston RJ Learman BS Zhang B Scheller EL Parlee SD Simon BR. Increased circulating adiponectin in response to thiazolidinediones: investigating the role of bone marrow adipose tissue. Front Endocrinol (Lausanne). (2016) 7:128. 10.3389/fendo.2016.00128 27708617 PMC5030308

[78] Wong CK Lit LC Tam LS Li EK Lam CW. Aberrant production of soluble costimulatory molecules CTLA-4, CD28, CD80, and CD86 in patients with systemic lupus erythematosus. Rheumatology (Oxford). (2005) 44:989–94. 10.1093/rheumatology/keh663 15870153

[79] Hock BD O'Donnell JL Taylor K Steinkasserer A McKenzie JL Rothwell AG. Levels of the soluble forms of CD80, CD86, and CD83 are elevated in the synovial fluid of rheumatoid arthritis patients. Tissue Antigens. (2006) 67:57–60. 10.1111/j.1399-0039.2005.00524.x 16451202

[80] Sakthivel P Shively V Kakoulidou M Pearce W Lefvert AK. The soluble forms of CD28, CD86 and CTLA-4 constitute possible immunological markers in patients with abdominal aortic aneurysm. J Intern Med. (2007) 261:399–407. 10.1111/j.1365-2796.2007.01773.x 17391115

[81] Ip WK Wong CK Leung TF Lam CW. Plasma concentrations of soluble CTLA-4, CD28, CD80 and CD86 costimulatory molecules reflect disease severity of acute asthma in children. Pediatr Pulmonol. (2006) 41:674–82. 10.1002/ppul.20432 16703581

[82] Wu H Chen J Song S Yuan P Liu L Zhang Y. β2-adrenoceptor signaling reduction in dendritic cells is involved in the inflammatory response in adjuvant-induced arthritic rats. Sci Rep. (2016) 6:24548. 10.1038/srep24548 27079168 PMC4832233

[83] Magnone M Leoncini G Vigliarolo T Emionite L Sturla L Zocchi E. Chronic intake of micrograms of abscisic acid improves glycemia and lipidemia in a human study and in high-glucose fed mice. Nutrients. (2018) 10:1495. 10.3390/nu10101495 30322104 PMC6213903

[84] Wang JS Lee WJ Lee IT Lin SY Lee WL Liang KW . Association between serum adipsin levels and insulin resistance in subjects with various degrees of glucose intolerance. J Endocr Soc. (2019) 3:403–10. 10.1210/js.2018-00359 30746502 PMC6364621

[85] Gómez-Banoy N Guseh JS Li G Rubio-Navarro A Chen T Poirier B. Adipsin preserves beta cells in diabetic mice and associates with protection from type 2 diabetes in humans. Nat Med. (2019) 25:1739–47. 10.1038/s41591-019-0610-4 31700183 PMC7256970

[86] Lo JC Ljubicic S Leibiger B Kern M Leibiger IB Moede T. Adipsin is an adipokine that improves β cell function in diabetes. Cell. (2014) 158:41–53. 10.1016/j.cell.2014.06.005 24995977 PMC4128197

[87] Choi J Kobayashi H Okuda H Harada KH Takeda M Fujimoto H. β-cell-specific overexpression of adiponectin receptor 1 does not improve diabetes mellitus in Akita mice. PLoS One. (2018) 13:e0190863. 10.1371/journal.pone.0190863 29304075 PMC5755906

