# Supplementary material for: Serum Concentration of the Phytohormone Abscisic Acid Is Associated With Immune-Regulatory Mediators and Is a Potential Biomarker of Disease Severity in Chronic Obstructive Pulmonary Disease
Source: Front Med (Lausanne). 2021 Jun 8;8:676058. doi: 10.3389/fmed.2021.676058 (PMC8217626; doi:10.3389/fmed.2021.676058)
Supplement: Supplementary file 1 [file Data_Sheet_1.PDF]

Supplementary Figures

Figure S1

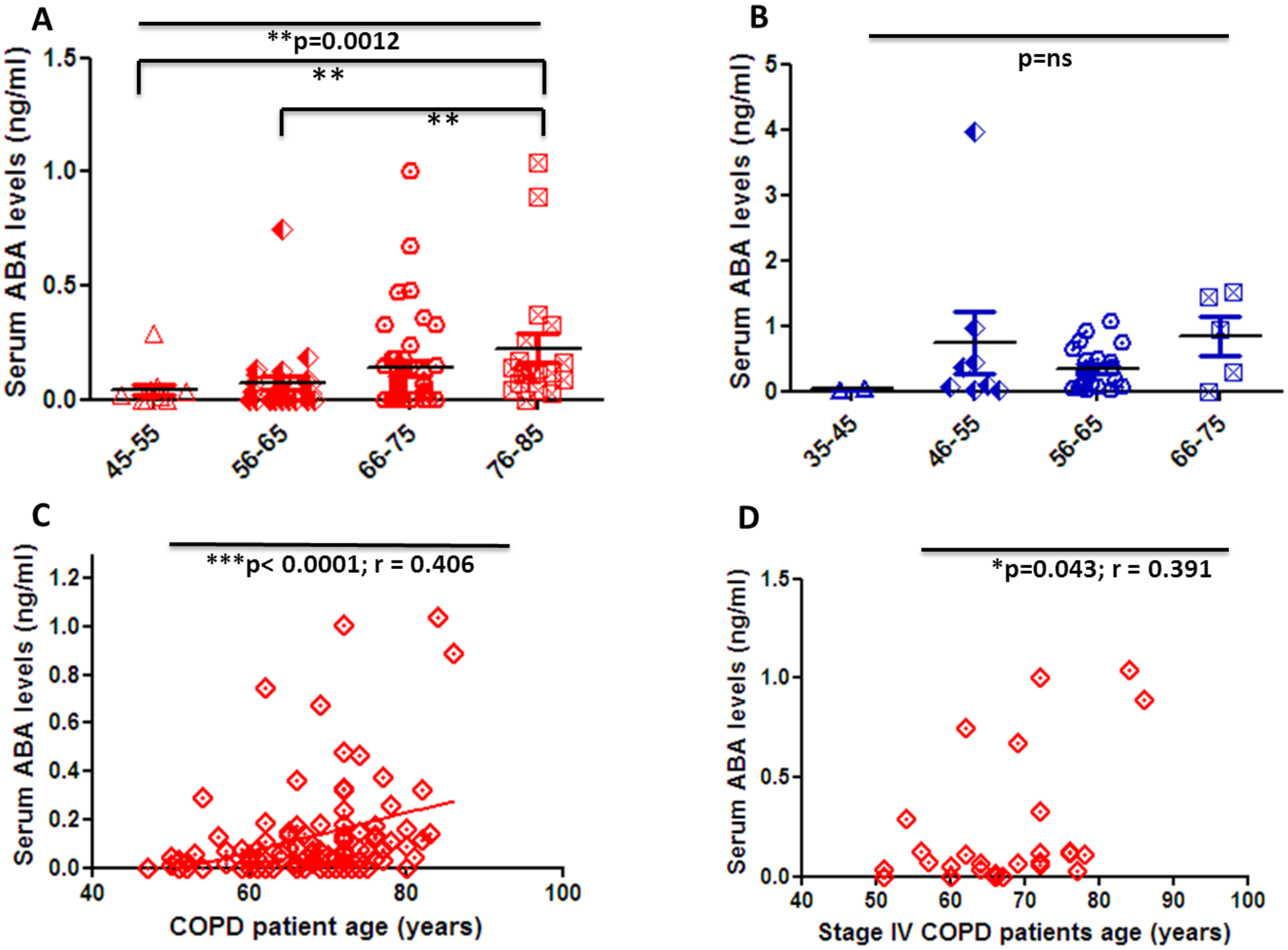

Figure S2

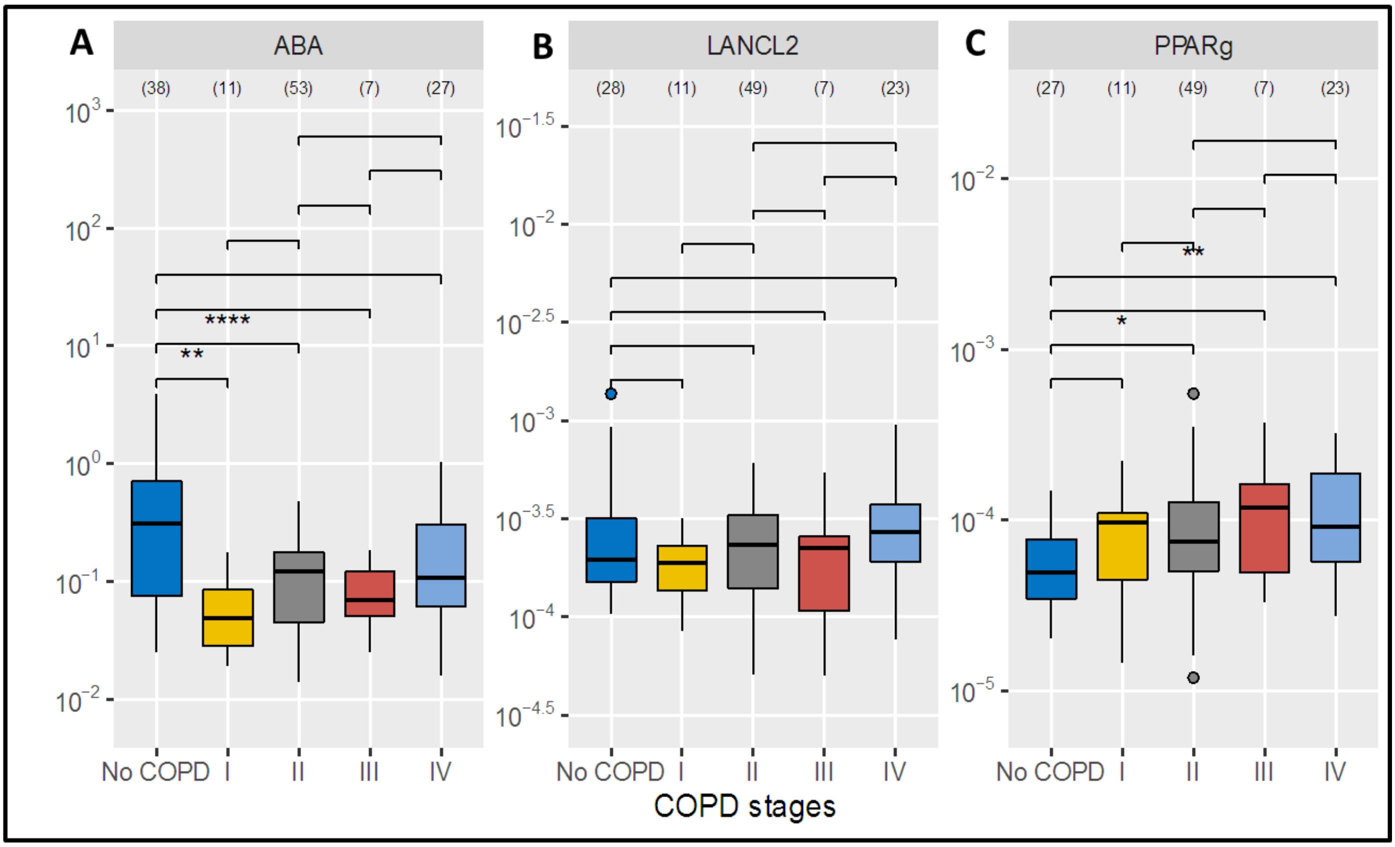
A
ABA
(38)
(11)
(53)
(7)
(27)
10^3
10^2
10^1
10^0
10^{-1}
10^{-2}
****
**
No COPD
I
II
III
IV
B
LANCL2
(28)
(11)
(49)
(7)
(23)
$10^{-1.5}$
10^{-2}
$10^{-2.5}$
10^{-3}
$10^{-3.5}$
10^{-4}
$10^{-4.5}$
No COPD
I
II
III
IV
COPD stages
C
PPARg
(27)
(11)
(49)
(7)
(23)
10^{-2}
10^{-3}
10^{-4}
10^{-5}
**
*
No COPD
I
II
III
IV

Figure S3

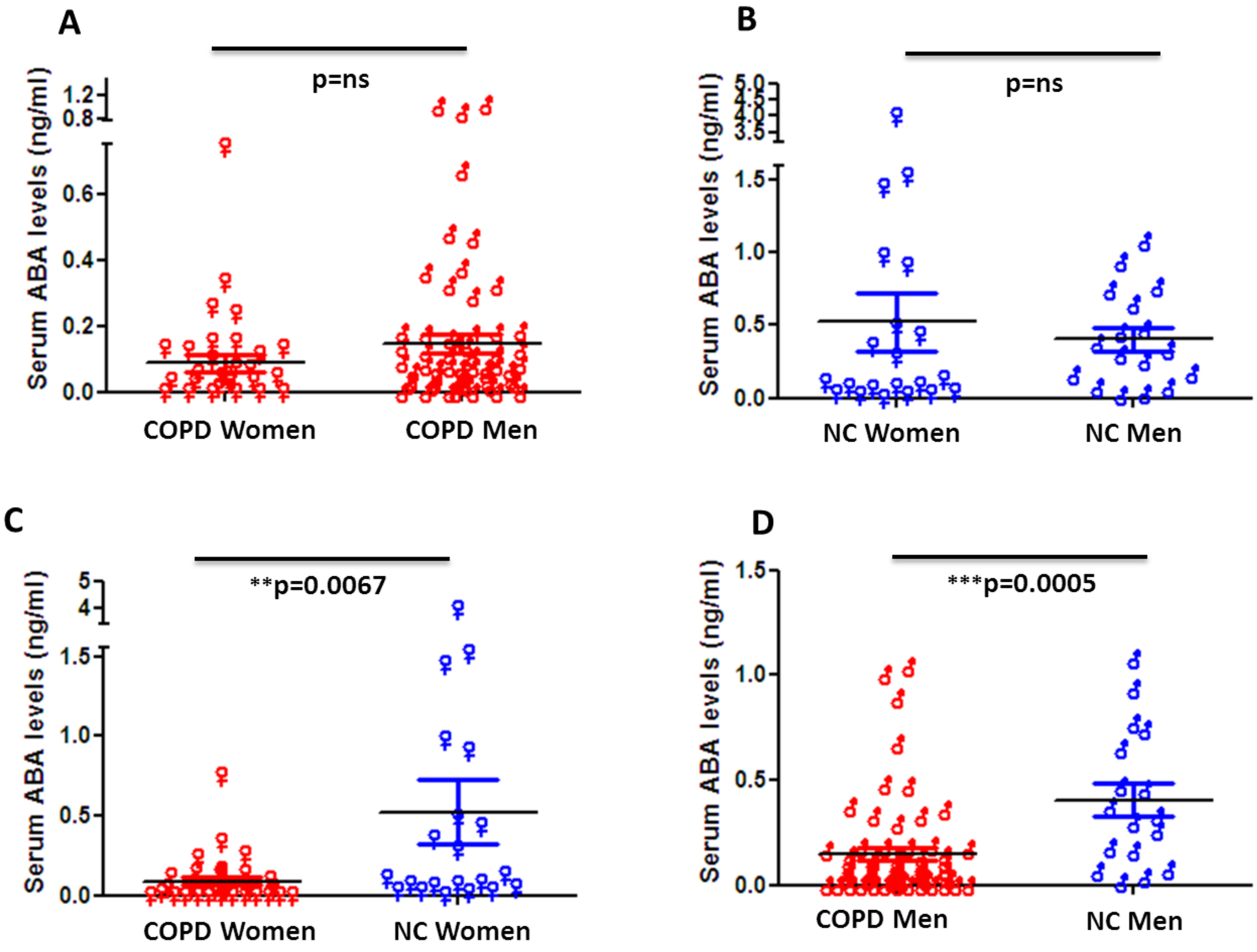
A
p=ns
Serum ABA levels (ng/ml)
1.2
0.8
0.6
0.4
0.2
0.0
COPD Women
COPD Men
B
p=ns
Serum ABA levels (ng/ml)
5.0
4.5
4.0
3.5
1.5
1.0
0.5
0.0
NC Women
NC Men
C
**p=0.0067
Serum ABA levels (ng/ml)
5
4
1.5
1.0
0.5
0.0
COPD Women
NC Women
D
***p=0.0005
Serum ABA levels (ng/ml)
1.5
1.0
0.5
0.0
COPD Men
NC Men

Figure S4

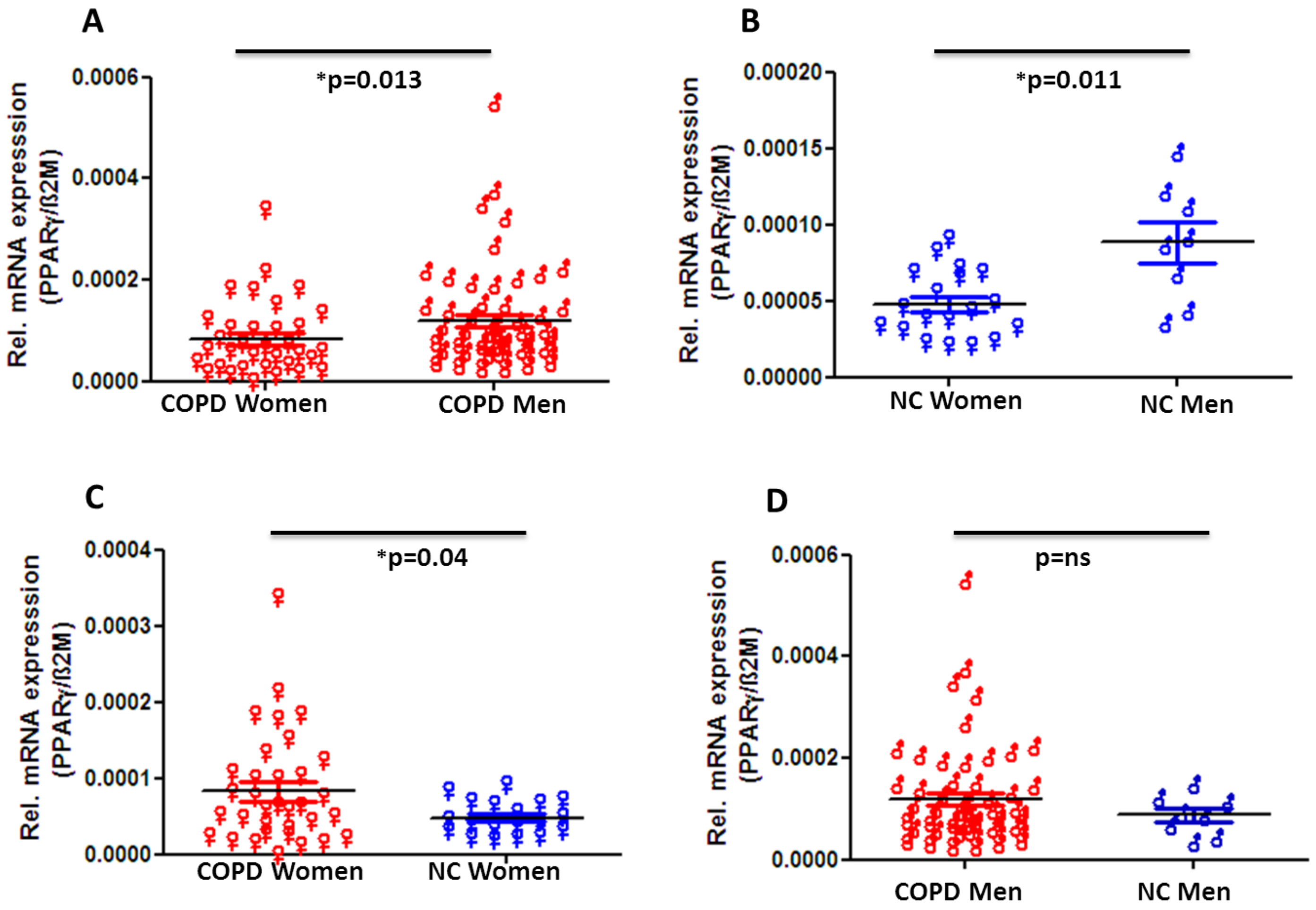
A
*p=0.013
Rel. mRNA expression (PPARγ/ß2M)
0.0006
0.0004
0.0002
0.0000
COPD Women
COPD Men
B
*p=0.011
Rel. mRNA expression (PPARγ/ß2M)
0.00020
0.00015
0.00010
0.00005
0.00000
NC Women
NC Men
C
*p=0.04
Rel. mRNA expression (PPARγ/ß2M)
0.0004
0.0003
0.0002
0.0001
0.0000
COPD Women
NC Women
D
p=ns
Rel. mRNA expression (PPARγ/ß2M)
0.0006
0.0004
0.0002
0.0000
COPD Men
NC Men

Figure S5

A

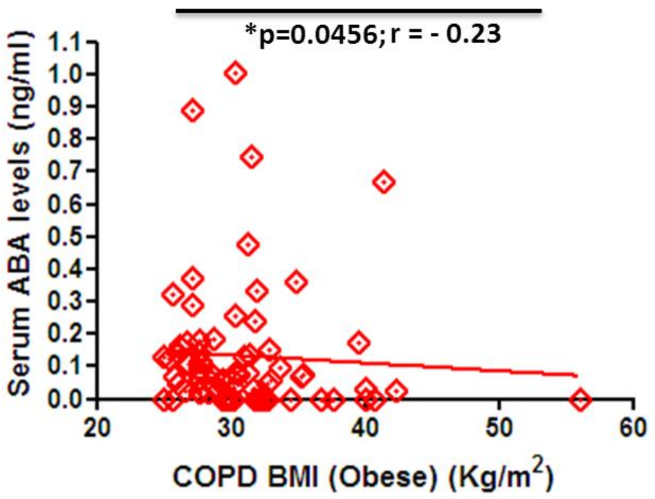
*p=0.0456; r = - 0.23
Serum ABA levels (ng/ml)
1.1
1.0
0.9
0.8
0.7
0.6
0.5
0.4
0.3
0.2
0.1
0.0
20
30
40
50
60
COPD BMI (Obese) (Kg/m2)

B

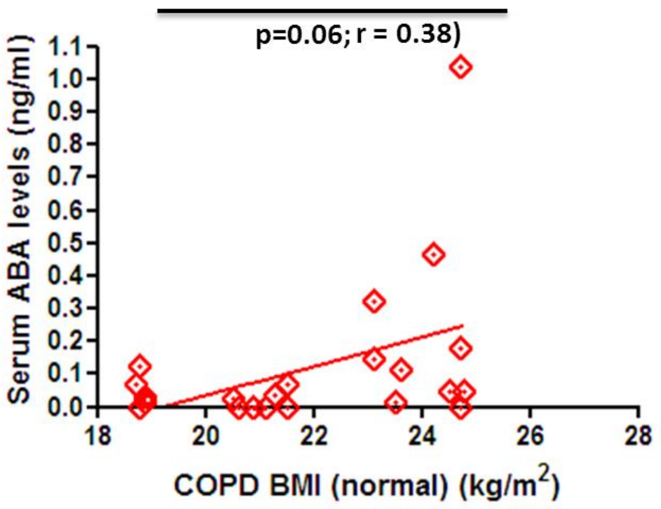
p=0.06; r = 0.38)
Serum ABA levels (ng/ml)
1.1
1.0
0.9
0.8
0.7
0.6
0.5
0.4
0.3
0.2
0.1
0.0
18
20
22
24
26
28
COPD BMI (normal) (kg/m2)

Figure S6

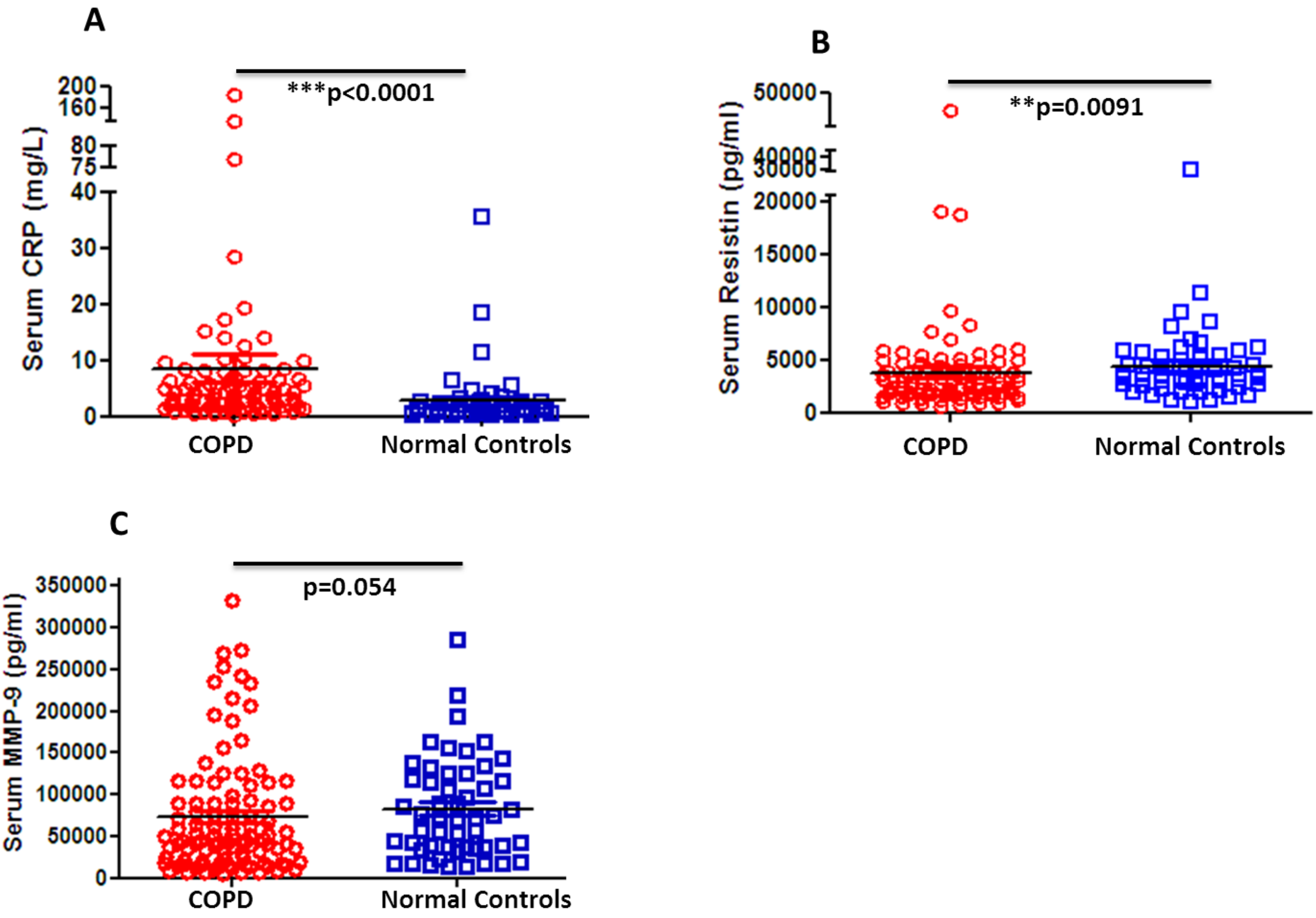
A
***p<0.0001
Serum CRP (mg/L)
200
160
80
75
40
30
20
10
0
COPD
Normal Controls
B
**p=0.0091
Serum Resistin (pg/ml)
50000
40000
30000
20000
15000
10000
5000
0
COPD
Normal Controls
C
p=0.054
Serum MMP-9 (pg/ml)
350000
300000
250000
200000
150000
100000
50000
0
COPD
Normal Controls

Figure S7

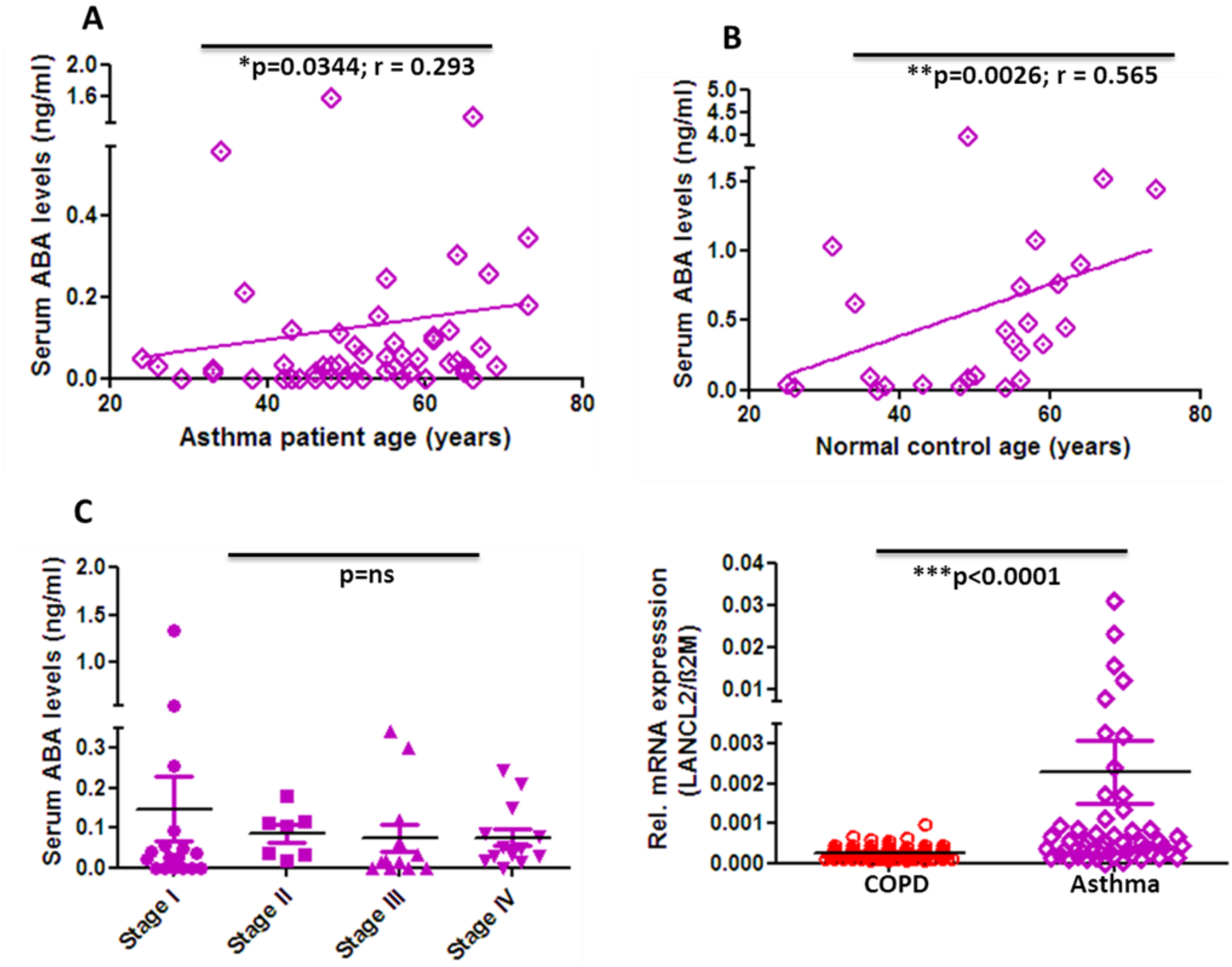
A
*p=0.0344; r = 0.293
Serum ABA levels (ng/ml)
Asthma patient age (years)
B
**p=0.0026; r = 0.565
Serum ABA levels (ng/ml)
Normal control age (years)
C
p=ns
Serum ABA levels (ng/ml)
Stage I
Stage II
Stage III
Stage IV
***p<0.0001
Rel. mRNA expresssion (LANCL2/ß2M)
COPD
Asthma

FIGURE LEGEND (Supplementary/ Supporting information)

Figure S1. Serum levels of ABA in COPD patients and controls when categorized based on age. COPD patients **(A)** and control subjects **(B)** classified based on their age, ranging from middle aged (45-55 and 56-65 years) to older adults (66-75 and 76-85 years). Correlation of serum ABA levels and **(C)** age of the COPD patients and **(D)** age of severely ill COPD patients (stage IV). The individual dots represent data obtained from individual subjects. Statistical analysis was done by either one way ANOVA using a Kruskal Wallis test with Dunn´s posthoc correction or non-parametric Mann-Whitney test. The correlation test was performed using non-parametric Spearman´s rank correlation coefficient test.

Figure S2. ABA serum levels and ABA receptor distribution by COPD stage progression (putting on a different y-axis scale for visualization purpose). Numbers in brackets represent sample size included in the test. The test statistics used are the ranksum pairwise test, adjusted for multiple comparison using Bonferroni approach. The tests shown are comparing the two groups connected by the lines below the p-value's notation (*p < 0.05, **p <0.01, ****p <0.0001)

Figure S3. **ABA serum levels in COPD patients and controls when categorized based on gender.** Circulating serum levels of ABA in **(A)** male and female COPD patients and **(B)** controls**.** Circulating serum levels of ABA in **(C)** women with COPD and control women and **(D)** men with COPD and control men. The individual dots represent data obtained from individual subjects. Statistical analysis was done by either non-parametric Mann-Whitney test or unpaired student *t* test.

Figure S4. Blood cell PPAR-γ gene expression of COPD patients and controls when categorized based on their gender. Blood cell PPAR-γ gene expression in male and female **(A)** COPD patients and **(B)** controls**.** Blood cell PPAR-γ gene expression in **(C)** women with COPD and control women and **(D)** men with COPD and control men. The individual dots represent data obtained from individual subjects. Statistical analysis was done by either non-parametric Mann-Whitney test or unpaired student *t* test.

Figure S5. Relationship between ABA serum levels and body mass index of COPD patients. Correlation of serum ABA levels in obese COPD patients and patients with normal body weight. The individual dots represent data obtained from individual subjects. Statistical analysis was done by using non-parametric Spearman´s rank correlation coefficient test.

Figure S6. Serum levels of COPD-associated clinical markers among COPD patients and controls. Circulating serum level of **(A)** CRP, **(B)** resistin, **(C)** MMP-9 in COPD patients and controls. The individual dots represent data obtained from individual subjects. Statistical analysis was done by either non-parametric Mann-Whitney test or unpaired student *t* test.

Figure S7. Relationship between ABA serum levels in asthmatics and controls and their age and serum levels of ABA among asthmatic disease severity. Correlation of ABA serum levels and age of **(A)** asthma patients and **(B)** controls. **(C)** Circulating serum levels of ABA among asthma patients when classified based on their disease severity. **(D)** Gene expression of LANCL2 on blood cells in COPD and asthma patients. The individual dots represent data obtained from individual subjects. Statistical analysis was done by either non-parametric Mann-Whitney test or unpaired student *t* test. The correlation test was performed using non-parametric Spearman´s rank correlation coefficient test.
